# Bacterial Community 16S rRNA Gene Sequencing Characterizes Riverine Microbial Impact on Lake Michigan

**DOI:** 10.3389/fmicb.2019.00996

**Published:** 2019-05-14

**Authors:** Cindy H. Nakatsu, Muruleedhara N. Byappanahalli, Meredith B. Nevers

**Affiliations:** ^1^Department of Agronomy, Purdue University, West Lafayette, IN, United States; ^2^Great Lakes Science Center, United States Geological Survey, Chesterton, IN, United States

**Keywords:** microbial communities, freshwater lake, 16S rRNA gene, hydrodynamic model, *Escherichia coli*

## Abstract

Restoration of degraded aquatic habitats is critical to preserve and maintain ecosystem processes and economic viability. Effective restoration requires contaminant sources identification. Microbial communities are increasingly used to characterize fecal contamination sources. The objective was to determine whether nearshore and adjacent beach bacterial contamination originated from the Grand Calumet River, a highly urbanized aquatic ecosystem, and to determine if there were correlations between pathogens/feces associated bacteria in any of the samples to counts of the pathogen indicator species *Escherichia coli*. Water samples were collected from the river, river mouth, nearshore, and offshore sites along southern Lake Michigan. Comparisons among communities were made using beta diversity distances (weighted and unweighted Unifrac, and Bray Curtis) and Principal Coordinate Analysis of 16S rRNA gene Illumina sequence data that indicated river bacterial communities differed significantly from the river mouth, nearshore lake, and offshore lake samples. These differences were further supported using Source Tracker software that indicated nearshore lake communities differed significantly from river and offshore samples. Among locations, there was separation by sampling date that was associated with environmental factors (e.g., water and air temperature, water turbidity). Although about half the genera (48.1%) were common to all sampling sites, linear discriminant analysis effect size indicated there were several taxa that differed significantly among sites; there were significant positive correlations of feces-associated genera with *E. coli* most probable numbers. Results collectively highlight that understanding microbial communities, rather than relying solely on select fecal indicators with uncertain origin, are more useful for developing strategies to restore degraded aquatic habitats.

## Introduction

Aquatic microbial contamination by pollutants derived from anthropogenic sources is a problem across the United States and worldwide; control of this contamination and restoration of degraded habitats can cost millions of dollars and considerable on-the-ground effort by water and land managers (Great Lakes Interagency Task Force, 2016; Steinman et al., 2017). Traditionally, indicator bacteria, such as *Escherichia coli* and/or enterococci, have been used to monitor potential

contamination of recreational waters (U.S. EPA, 1986). Often, these bacteria are not adequate to identify contamination sources because they can originate from a variety of warm-blooded animals and from environmental sources (Byappanahalli and Ishii, 2011). This lack of specificity has led to the development of other methods, such as detection of source-host bacterial indicators (see Field and Samadpour, 2007).

Microbial source tracking has been used in recent years to identify specific sources of fecal contamination through the use of targeted genetic markers (Harwood et al., 2014). Genetic markers have been used to indicate microbial contamination from humans, birds, dogs, and other animals (Harwood et al., 2014). This targeted approach is useful for identifying and mitigating microbial contamination if there is a dominant contamination source, but restoration becomes more complicated if there are multiple sources (Byappanahalli and Ishii, 2011; Nevers et al., 2014).

With the uncertainty associated with indicator bacteria and microbial source tracking, as well as the need to refine specificity of source identification, particularly in instances of legal obligation, better characterization of pollutant sources contributing to fecal contamination and associated links to sources is needed. In the past 2–3 decades, molecular techniques targeting the 16S rRNA gene and other genetic markers have been developed to characterize and analyze microbial communities from a variety of habitats including soil and water (Konopka et al., 1999; Nakatsu, 2007; Tanaka et al., 2014). More recently, the decrease in cost of next-generation high throughput sequencing technology has enabled the use of metagenomic approaches (targeted and non-targeted) to differentiate sources of aquatic microbial contamination (Newton et al., 2013; Cloutier et al., 2015; Newton et al., 2015). The depth of information acquired by using these advanced molecular genetic approaches provides a means to characterize microbial composition, distribution, and transportation pathways in the environment and to relate them to understand pollution mechanisms (Newton et al., 2013; Halliday et al., 2014).

Through federal programs (e.g., The Beach Act of 2000; Great Lakes Restoration Initiative, 2009-current; International Joint Commission, 2012), federal-state partnerships have been established to decrease contamination sources and the effects of contamination on these lake ecosystems; among these contamination concerns are beach closings due to high concentrations of indicator bacteria such as *E. coli*. The objective of this study was to determine if samples with elevated levels of *E. coli* were correlated with potential pathogens or other fecal indicator bacteria in the microbial community. A 16S rRNA gene targeted high throughput sequencing approach was used to determine microbial community structure and composition. The objectives were (1) to determine the similarity of nearshore and offshore lake microbial communities to the adjacent riverine water source and (2) to determine the incidence and correlation of pathogens/feces associated species in the aquatic microbiome to the pathogen indicator species *E. coli* counts. The results will help to illuminate the association of shoreline and riverine bacterial communities and the potential contribution of bacteria originating from the Grand Calumet River in northern Indiana. The outcome of this work will contribute to determining the critical role of microbial communities in these degraded ecosystems and to aid in developing and assessing effective strategies for management and restoration of these environments.

## Materials and Methods

### Study Area

The study area is located in northern Indiana along the southern shore of Lake Michigan of the Laurentian Great Lakes. The focal point of the area is the Grand Calumet River, which has been highly urbanized during industrialization of the early 20th century. The Grand Calumet River flows into Lake Michigan through the channelized Indiana Ship Canal, and the entire river and associated shoreline is considered an “Area of Concern” by the International Joint Commission on boundary waters between the United States and Canada and therefore the focus of significant restoration efforts.

### Sample Collection

**FIGURE 1:**
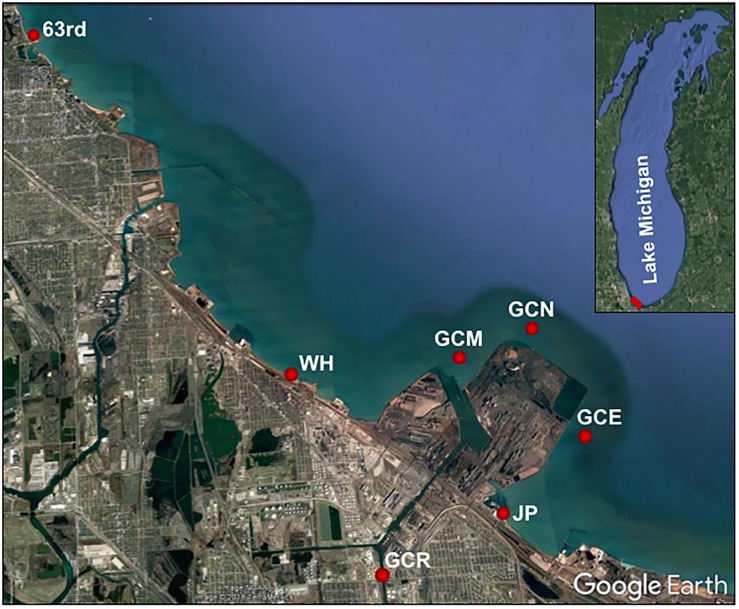
Sampling locations along the southern shore of Lake Michigan. Latitude and longitude for the study locations are as follows: 63rd, 41.782209/–87.572926; WH, 41.685118/–87.492282; GCR, 41.6394/–87.471276; GCM, 41.68719002/–87.43974003; GCN, 41.69217/–87.41554995; GCE, 41.66712/–87.40494006; and JP, 41.650478/–87.433551.

Sampling sites were located in the Grand Calumet River at Columbus Drive (GCR), at the mouth of the river (GCM), at offshore locations north (GCN) and east (GCE) of the peninsula that lies between the river mouth and Jeorse Park, and at three nearshore locations: Jeorse Park (JP), East Chicago, IN; Whihala Beach (WH), Whiting, IN; and 63rd Street Beach (63rd), Chicago, IL (Figure 1; Byappanahalli and Nevers, 2019).

Water samples (∼1.5 L) were collected in triplicate during three independent events in the summer of 2015. Two dates were during dry weather conditions (8/12, 9/1) and one date was after a rainfall event (9/21). The seven locations represented four sources: river (9 samples), river mouth (9 samples), nearshore (27 samples), and offshore (18 samples), for a total of 63 samples. Water from GCR was collected by tossing a sterile collection bucket from the bridge crossing at Columbus Drive; offshore surface water samples (GCM, GCN, GCE) were collected from a boat by dipping a sterile 1-L collection bottle below the surface; and nearshore samples (JP, WH, 63rd) were collected by dipping a 1-L collection bottle below the surface in 45-cm deep water. All samples were stored on ice directly after collection for return to the laboratory for processing within 6 h of collection.

### DNA Extraction

Upon return to the laboratory samples were concentrated by vacuum filtration first through a 5.0 μm nitrocellulose (Millipore) then 0.2 μm nitrocellulose filter (Millipore) then stored at −80°C until DNA extraction. Total genomic DNA from each 0.2 μm filter was extracted using the MoBio PowerWater kit according to the manufacturer’s instructions. Nucleic acid quality (i.e., 260/280 ratio) was measured with a Nanodrop 1000 spectrophotometer (Thermo Fisher Scientific, Wilmington, DE, United States). DNA concentrations for all samples were measured by fluorometric quantitation using a Qubit^®^ instrument and High Sensitivity dsDNA HS Assay kit (Thermo Fisher Scientific, Waltham, MA, United States); and purified DNA extracts were stored at −80°C until used.

### Illumina 16S rRNA Gene Sequencing

The 16S rRNA gene in water DNA extracts was PCR-amplified using primers targeting the V3-V4 region (343-forward TAC GGR AGG CAG CAG and 804-reverse CTA CCR GGG TAT CTA ATC C) (Liu et al., 2008; Nossa et al., 2010). Primers with dual index tags were used to differentiate multiple samples in a single run following the manufacturer’s (Illumina, San Diego, CA, United States) suggested step out protocol (Gloor et al., 2010). Reactions were carried out using ∼10 ng of template DNA in Q5^®^ High Fidelity DNA Polymerase 2X master mix (New England Biolabs). PCR amplicons were purified using AxyPrepMag PCR clean-up kit (Axygen Scientific) and quantified using a Nanodrop 3000 fluorospectrophotometer after staining with the QuantiFluor dsDNA System (Promega). Equimolar amounts of amplicons from each sample were combined and sent to the Purdue Genomics Core Facility for 2 × 250 paired end sequencing using a MiSeq Illumina system. Sequence reads were pre-processed to remove primer tags and low-quality sequences, and paired end reads were merged using PANDAseq software (Masella et al., 2012).

### 16S rRNA Gene Sequence Analysis

Sequences were analyzed using the QIIME pipeline (version 1.9.1) (Caporaso et al., 2010). Operational taxonomic units (OTUs) were picked using the “pick_open_otus” option in QIIME (Rideout et al., 2014) that uses a 97% sequence similarity threshold, the uclust method (Edgar, 2010) for clustering, sequence alignment using PyNAST (Caporaso et al., 2010), and taxonomic assignment to the Greengenes data set version 13_5 (McDonald et al., 2012) using the RDP classifier (Wang et al., 2007). The lowest number of reads among the samples, 24,890, was chosen to rarefy datasets to use equal number of reads for all community comparisons. Beta-diversity measures were calculated using phylogenetic Unifrac distances (weighted and un-weighted) (Lozupone et al., 2011) and non-phylogenetic distance (Bray Curtis). Alpha-diversity measurements were used for richness and evenness (Shannon diversity), richness (ChaoI index, observed-species), Faith’s phylogenetic diversity (PD whole tree) and Good’s coverage to assess the completeness of OTU representation in each sample. Venn diagrams illustrating genera common to all samples was produced using the Venny program (Oliveros, 2007/2015).

The SourceTracker (Knights et al., 2011) plugin in QIIME 1.9.1 was used to predict if river samples were significant contributors of OTUs to offshore or nearshore sites. Default conditions of the program were used after filtering out OTUs that were present in less than 1% of samples. This method used relative proportion of genera present to estimate the probability that the river was a significant source of microbes into the lake.

### Quantification of Indicator Bacteria

Water samples were analyzed for *E. coli* using the IDEXX Colilert-18 and Quanti-Tray 2000 method (IDEXX Laboratories, Westbrook, Maine), a defined substrate technology (Edberg et al., 1991). Generally, 100 ml of water was analyzed; excessively turbid samples were diluted as needed before analysis. Results are calculated as most probable number (MPN)/100 mL (Byappanahalli and Nevers, 2019).

### Hydrometeorological Measurements

Ambient conditions were measured at the time of sample collection: water and air temperature (°C, H-B Instrument, Trappe, PA, United States), current speed (U.S. EPA, 2008) and direction (eastward, westward, float method), wind direction and speed (m/s, SKYTECH, Weatherhawk, Logan, UT, United States), wave height (inches, meter stick), rainfall ( <24 h, <48, <72, and >72 prior to sample collection), and cloud cover (percent scale). Bird counts were recorded, as well as general beach conditions, including amount of debris, trash, and *Cladophora* algae. Water samples were analyzed for turbidity in the laboratory (2100N, Hach, Loveland, CO, United States) (Byappanahalli and Nevers, 2019).

Hydrologic data, including water temperature (°C), specific conductance (μs/cm), pH, DO (mg/L), and turbidity (FNU) at East Chicago, IN (Jeorse Park, 04092788) and Jackson Park, IL (63rd, 04092440) were extracted from the United States Geological Survey National Water Information System (2018) database for use in the analysis (United States Geological Survey, 2018).

In addition, at 63rd, hydrometeorological data were collected from a multi-parameter weather station (Vaisala WXT520) installed on a light post (30’) at the beach and a data buoy (NexSens CB-100) installed in the swimming area (∼1.5 m depth). Data obtained from the weather station included measurements of wind direction and speed (m/s), air temperature (°C), rainfall (cm), solar radiation (LI-COR sensor; LI-200), relative humidity (%), and barometric pressure (mm/Hg). Data obtained from the buoy included turbidity (NTU, FTS sensor, DTS-12), wave height (m) and wave period (seconds) (NexSens Accustage pressure transducer; OEM, Keller America), and water temperature (°C).

### Statistical Analyses

Significant differences in beta diversity among communities were determined using 999 permutations of PERMANOVA (Anderson, 2001); then, PERMDISP (permutational analysis of multivariate dispersions) (Anderson, 2006) was used to ensure significant differences were not due to differences in dispersion. Differences in alpha diversity metrics were determined using non-parametric *t*-tests with 999 permutations. Additionally, potential biomarkers differentiating collection sites was determined using LEfSE (linear discriminate analysis effect size) (Segata et al., 2012). LEfSE is a three-step process in which non-parametric Krustal Wallis is performed to first identify taxa that differed significantly among sources, then for these taxa, pairwise Wilcoxon rank-sum test is performed and finally linear discriminant analysis (LDA) is used to estimate the effect size and biological consistency within groups being tested. Data output are taxa with LDA greater than 2.0 at any taxonomic level that is discriminating.

Correlations between genera and *E. coli* contamination levels were determined using Spearman’s Rho. Associations among relative abundances of genera in each sample and all measured environmental factors (water temperature, air temperature, turbidity, dissolved oxygen, and *E. coli* MPN) as well as water source (river, river mouth, nearshore, or offshore) were determined using canonical correspondence analysis (CCA) (Ter Braak, 1986; Ter Braak and Verdonschot, 1995). Significantly different correlations were calculated using a Monte Carlo test with 999 permutations. Hydrometeorological data were sub-divided into: 1, low; 2, medium; 3, medium/high; and 4, high for analysis. This was accomplished using the visual binning method in SPSS version 23 (SPSS, 2014); cut points for binning were made using the ±1 SD option. All other statistics were performed using the Paleontological Statistics package version 3.01 (PAST software^1^) or software available in QIIME. Differences were considered significant if *p* ≤ 0.05 with multiple comparisons using 999 permutations.

## Results and Discussion

One of the primary needs before initiating beach restoration for recreational use is to determine sources of fecal contamination in nearshore areas. Depending on the findings, remedial strategies can then be employed to reduce or mitigate those sources contributing to water quality; for instance, gull deterrence using trained dogs and physical modifying structures (e.g., breakwalls) to improve water circulation and dissipation of contaminants at shallow, embayed beaches are a few examples of management actions to restore water quality (Ge et al., 2012; Nevers et al., 2018).

The sampling locations for this study were specifically chosen because the shorelines had traditionally suffered from elevated levels of fecal indicator bacteria (*E. coli*) leading to frequent beach closures (Byappanahalli et al., 2015; Nevers et al., 2018). Specifically, the Grand Calumet River and associated shoreline is an area designated for intensive restoration efforts under a bi-national agreement between the United States and Canada (International Joint Commission, 2012). Nearshore water quality at Jeorse Park has steadily deteriorated between 2005 and until recently, as evidenced by increased *E. coli* levels exceeding the state recreational water quality standard for safe swimming (Byappanahalli et al., 2015; Nevers et al., 2018). Early efforts in source tracking have identified shorebirds and human fecal contamination at these beaches. Unknown are the relative inputs of point (river) and other non-point (shoreline) sources.

Illumina sequencing results produced an abundant number of reads for bacterial communities across the sites. The 63 water samples collected from seven locations on three dates produced a total of 5,136,926 paired-end reads after quality filtering and merging. There was an average of 81,538 ± 63,981 reads per sample, ranging from 7,050 to 396,284 reads. The sample with the lowest read (a river sample from the first sampling date) was excluded from community analyses, and the remaining 62 samples were rarified to 24,890 reads per sample, with a Goods coverage of 0.96 (±0.01 SD), ranging from 0.94 to 0.98.

### Alpha Diversity Differences Among Sites

**FIGURE 2:**
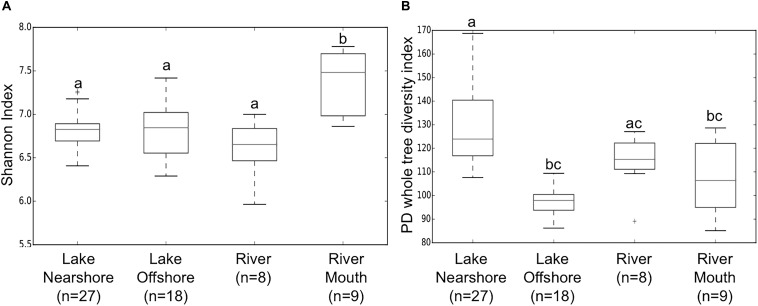
Boxplots of means and SD (standard deviations) of within sample (alpha) diversity indices **(A)** Shannon (richness and evenness) and **(B)** PD whole tree (Faith’s Diversity, phylogenetic richness) of samples from different water sources. Non-parametric two sample *t*-test with 999 permutations with significant differences *p* < 0.02 illustrated by different letters above bars.

In terms of alpha diversity, rarefaction curves indicated Shannon diversity reached saturation in all samples (Supplementary Figure S1A) and was beginning to level off using other indices (e.g., phylogenetic diversity, PD whole tree Supplementary Figure S1B). The Shannon index, a measure of richness and evenness, was statistically higher for river mouth samples compared to the other sources (Figure 2A). Whereas, PD whole tree, which only accounts for species richness, indicated the phylogenetic diversity was statistically higher in lake nearshore samples compared to lake offshore, river and river mouth samples (Figure 2B). This could indicate more complex input sources to the nearshore community because of coastal processes likely mediated by (a) hydrometeorological events, previously seen for *E. coli* in nearshore waters (Ge et al., 2012), (b) exchanges between shoreline sources, and (c) interactions between nearshore and offshore communities.

### Beta Diversity Differences Among Sites and Dates

**FIGURE 3:**
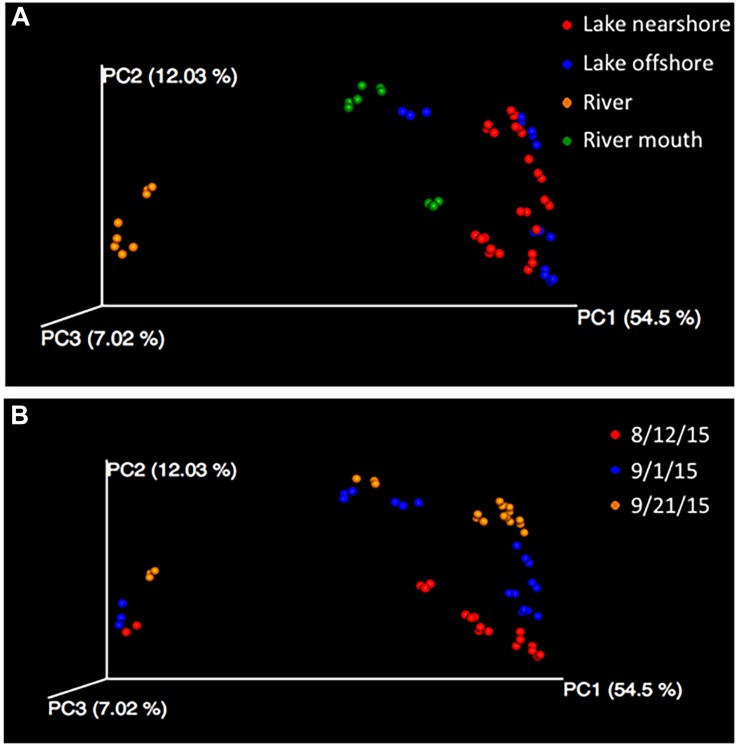
Principal coordinate analysis (PCoA) of Bray Curtis dissimilarity metrics of samples labeled according to the **(A)** water sources and by **(B)** sampling date. Differences among microbial communities are significant, PERMANOVA and ANOSIM *P* = 0.001 and meets the assumption of dispersion homogeneity because PERMDISP is not significant.

Beta diversity analysis of the 16S rRNA gene sequences indicated that the bacterial community in the Grand Calumet River was the least similar to the communities along the shoreline or offshore. The PCoA plots of beta diversity distances among samples from the four water sources illustrate that the main separation was between the river samples and all other sources (Figure 3A). The first three axes of Bray Curtis distances accounted for ∼74% of the variation with the river samples separated from the other sources along the first axis (PCoA 1 = 54.5%). This difference was statistically significant based on PERMANOVA (*P* = 0.001) and ANOSIM (*R* = 0.598, *P* = 0.001) and not significant due to dispersion (PERMDISP *P* = 0.41). If the samples were differentiated into the seven sampling locations, the differences remained significant. Using distances based on phylogenetic relationship of community members (weighted and unweighted Unifrac) yielded similar statistically significant results (data not shown). Similarity between samples collected at the mouth of the river and offshore samples indicates that perhaps river flow is minimal, with an extensive mixing zone in the river mouth. This suggests that during the three sampling periods between August and September 2015, bacterial contribution from the river to the lake was minimal. These findings support previous research that much of the Jeorse Park (JP) nearshore microbial contamination, which has led to recreational beach closures, is from local, non-point sources (e.g., birds, *Cladophora* algae) with few anthropogenic contributions (e.g., combined sewer outflows) from the river (Byappanahalli et al., 2015; Nevers et al., 2018). This indicates that mitigation strategies for this shoreline will likely differ from those initiated for other riverine habitats in the Grand Calumet River corridor.

The date of sampling was also significantly different (*P* = 0.001) although community differences were less pronounced than between water types (Figure 3). Factors that differed by sampling dates were rain events, temperature and dissolved oxygen (Supplementary Table S5). While collection dates targeted one rain event (9/21) to compare it to two dry weather events, there was no difference in collection dates that could be attributed to rain rather than the other factors that differed among the three dates. Interestingly, combined sewer overflow was recorded at the Hammond Sanitary District on 9/21^2^, but the volume of water released was likely insufficient to impact the shoreline microbial communities. The similarity of community composition in the river between sampling dates and dissimilarity with the shoreline communities may result from a combination of frequent combined sewage overflows and low river flow, respectively.

### Microbial Taxa Represented

There were 50 phyla (Supplementary Table S1) represented in the 16S rRNA gene sequences, and >90% of the relative abundance could be attributed to three phyla: Proteobacteria (45.6 ± 5.9%), Actinobacteria (26.8 ± 8.9%), and Bacteroidetes (22.8 ± 6.1%) (mean ± SD). These findings are consistent with other studies suggesting the dominance of these three phyla in freshwater systems (Newton et al., 2011; Mou et al., 2013). Among the remaining phyla, those representing >0.1% included the Chloroflexi (0.9 ± 0.9%), Cyanobacteria (1.5 ± 1.3%), Firmicutes (0.3 ± 0.3%), and Verrucomicrobia (0.7 ± 0.6%); 0.9 ± 0.4% was not assigned to a phylum. Of the remaining phyla, 25 represent currently tentative phyla likely lacking in cultivated isolates needed for classification. Dominance of a few taxa was found at all taxonomic levels of classification. For example, there was a total of 786 genera assignments, of which 23 (∼3% of total genera) had a mean relative proportion >1% (mean of all samples) and accounted for 85.6 ± 4.0% of the community. The majority, 505 (64%), could not be assigned to a genus or is currently listed as a candidate genus.

One of the major advantages of using targeted metagenomic techniques, such as the 16S rRNA gene sequencing, is that they are culture-independent and can theoretically recover almost all bacterial taxa in any habitat. However, despite rapid advancements in this area, most bacterial species in communities remain to be identified (Locey and Lennon, 2016). A general lack of cultured organisms with sequencing information (in NCBI and other databases) essentially limits taxonomic identification from the sequenced data.

### Taxa Differences Among Sites

**FIGURE 4:**
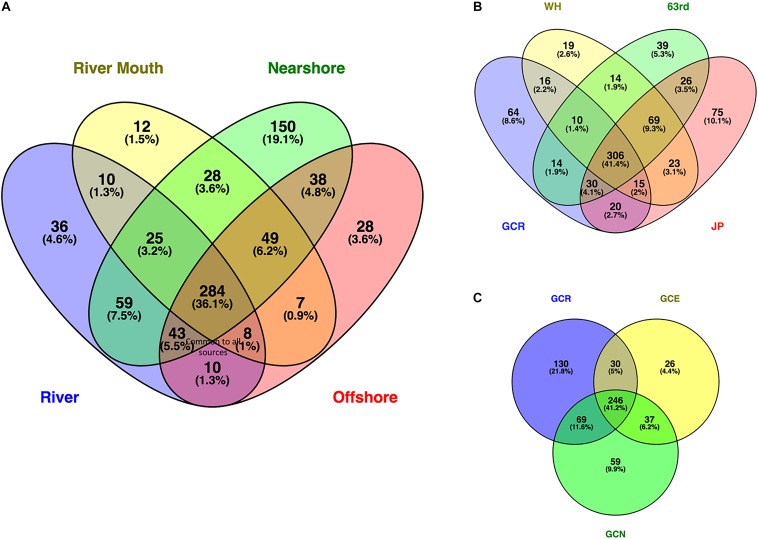
Venn diagram illustrating shared and unique genera among **(A)** water sources, **(B)** river and nearshore sites, and **(C)** river, river mouth, and lake offshore sites. Sampling sites in **(A,B)** represented by nearshore locations Jeorse Park (JP), Whihala beach (WH), and 63rd Street Beach (63) in Chicago, Grand Calumet River (GCR), mouth of the river (GCM), offshore sites north (GCN) and east (GCE). Calculations and illustrations were determined using the Venny program (Oliveros, 2007–2015).

Venn diagrams illustrate the total number of genera shared among samples as well as the percentage of the total number of genera. About one third (284 genera, 36.1%) of the total genera identified were common to all samples (Figure 4A). They represent an average of more than 60% of the relative proportion of genera in these samples. Shared genera, such as Actinobacteria ACK-M1, likely represent those common to aquatic ecosystems (Newton et al., 2011; Mou et al., 2013). Only an additional 1.3% more genera were common to the river and river mouth samples only (Figure 4A), indicating there are very few unique genera from the river flowing into the lake microbial community, perhaps because of low river flow and minimal mixing. The combined nearshore sites had the greatest number of unique genera (19.1%). A comparison of the number of genera common among the river and individual nearshore sampling sites (Figure 4B), and river and offshore sites (Figure 4C) showed 41.4 and 41.2%, respectively, were shared. The nearshore sites had unique genera ranging from 2.6 to 10.1%. This could be the result of high variation among the nearshore collection sites: 63rd (an urban beach) is located much further north of the other two sites, and JP and WH are situated on opposite sides of a large constructed industrial peninsula. There are likely different sources of microbial communities, potentially arising from beach sand (Solo-Gabriele et al., 2016; Staley and Sadowsky, 2016) and the nuisance shoreline alga *Cladophora* (Zulkifly et al., 2012; Whitman et al., 2014a; Chun et al., 2017), and the relative locations of 63rd and JP further isolates exchange along the shoreline (Ge et al., 2012; Byappanahalli et al., 2015).

### Potential for Grand Calumet River to Contribute to Nearshore Microbial Communities

Since the Venn diagrams only illustrated the presence and absence of genera at each study site, additional analysis was conducted using SourceTracker that accounts for the relative proportions of each OTU. SourceTracker estimated that the highest possible contributions of river water to the river mouth, offshore, and nearshore (combined) (Table 1) or nearshore sites, 63rd, WH, JP (individually) (Table 1) were on the first sampling date (8/12/15). Krustal Wallis test indicated that there was an overall significant difference (*p* < 0.002) in contributions from the river; however, pairwise comparisons only showed that nearshore was significantly different from river mouth and offshore. Analysis specifically of the river to nearshore sites indicated significantly higher contribution to 63rd on 8/12/15; alternately, 63rd and WH had significantly lower contribution than JP on 9/1/15 (Table 1).

**TABLE 1:** Estimation of Grand Calumet River as a potential source^†^ of bacterial populations (mean percentages ± standard error of mean) on three sampling dates for (A) Lake communities as sink^‡^, or (B) Specific nearshore communities as sink^§^.

	**A**			**B**		
**Date**	**River mouth**	**Lake nearshore**	**Lake offshore**	**JP**	**WH**	**63**
8/12/15	83.7 ± 0.4%^ab^	81.1 ± 1.1%^a^	76.2 ± 0.9%^ab^	79.6 ± 1.5%^ab^	79.9 ± 2.2%^ab^	84.0 ± 1.0%^a^
9/1/15	80.7 ± 0.4%^ab^	71.9 ± 0.8%^b^	73.5 ± 2.1%^ab^	74.2 ± 0.9%^ab^	69.4 ± 1.1%^b^	72.0 ± 0.6%^b^
9/21/15	79.3 ± 0.3%^b^	78.7 ± 0.7%^a^	76.0 ± 0.8%^ab^	77.1 ± 0.3%^ab^	77.4 ± 0.3%^ab^	81.6 ± 0.4%^ab^

*^†^Source contribution estimated using SourceTracker (Knights et al., 2011) plugin in QIIME 1.9.1 (Caporaso et al., 2010) using default conditions. ^‡^Sinks are river mouth, nearshore, and offshore samples a (*n* = 3, 9, 6 per sampling date, respectively), Krustal Wallis (*P* = 0.00003). ^§^Sinks are three nearshore beaches: Jeorse Park (JP), Whihala (WH), and 63rd Street (63) in Chicago, Krustal Wallis (*P* = 0.004). Different superscripts letters in the tables indicate significant difference of pairwise comparisons by date using Dunn’s *post hoc* test with Bonferroni correction.*

**FIGURE 5:**
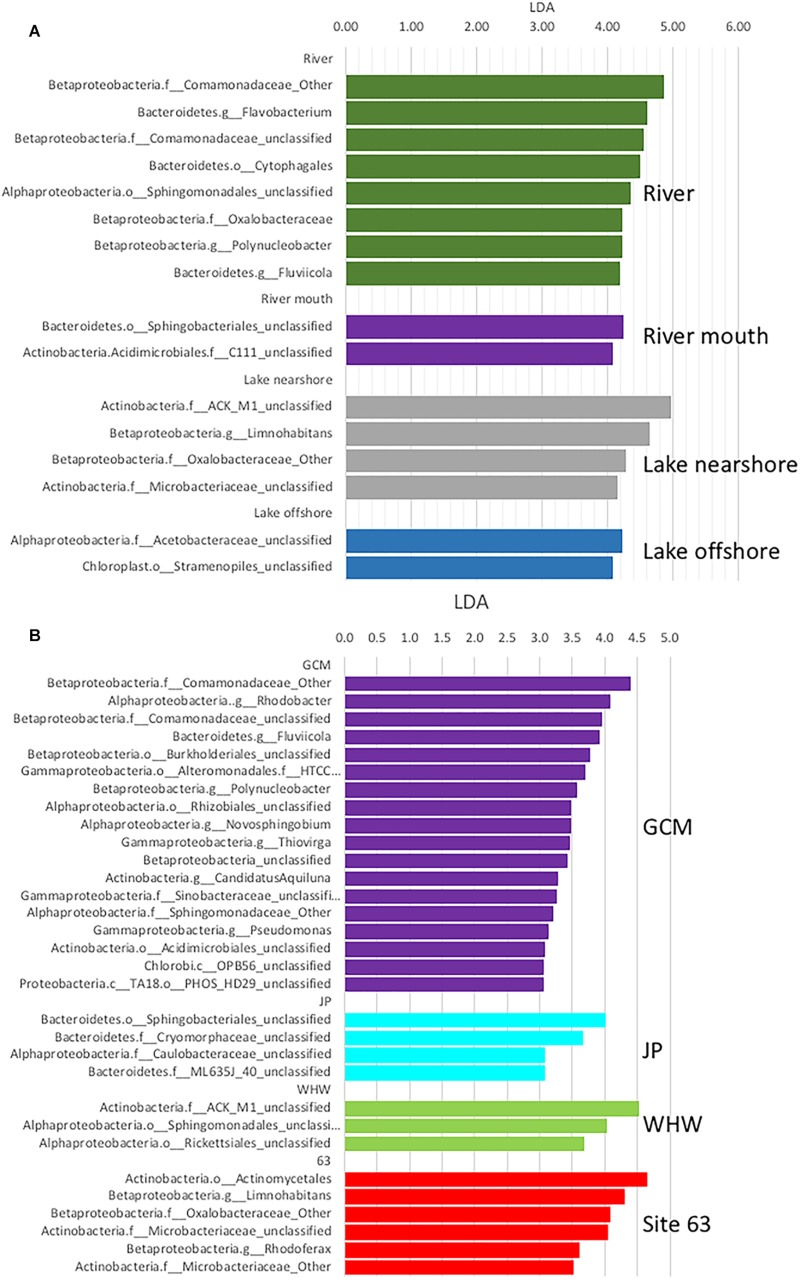
Linear Discriminate Analysis Effect Size (LEfSE) analysis of microbial communities from the various **(A)** water sources and **(B)** nearshore collection sites and river mouth. Only taxa that differed significantly (*p* < 0.05) with LDA effect sizes greater than 4.0 in **(A)** and 3.0 in **(B)** are illustrated in the histograms. Taxa names include phylum followed by lowest taxonomic assignment available. Nearshore beaches include Jeorse Park (JP), Whihala (WH), and 63rd Street (63) in Chicago, and mouth of the Calumet River (GCM).

The program LEfSE was used to identify the taxa that differed among the different sampling sites and to determine if any of these genera were potentially pathogens or indicators of potential fecal contamination. LEfSE analysis identified significant differences in taxa among sample sources (river, nearshore and offshore) at all levels of taxonomic classification, and also in comparisons of sampling sites and sampling dates. Comparisons of sample sources (river, nearshore and offshore) indicated there were 233 taxa with LDA effect sizes greater than 2.0 (Supplementary Table S2). The most taxa differences were in the river samples (96 taxa) followed by river mouth (63 taxa), nearshore (55 taxa) and offshore (42 taxa). Examination of taxa with LDA effect sizes greater than 4.0 at the lowest level of classification showed that most belonged to taxa yet to be classified (Figure 5A). Taxa that could be classified to the genus level were *Flavobacterium, Polynucleobacter*, and *Fluviicola* in the river samples and various unclassified taxa in the other water sources (Figure 5A); *Polynucleobacter* has been shown to be widespread in streams associated with human/anthropogenic activities (Hosen et al., 2017).

When the sources were split into specific sites there were 313 taxa with LDA effect sizes greater than 2.0 (Supplementary Table S3). The most taxa differences were in the river samples (84 taxa) followed by the JP nearshore site (76 taxa), river mouth (63 taxa), then nearshore site 63 (36 taxa), offshore site GCE (34 taxa), GCN (12 taxa) and the nearshore site WH (9 taxa). In this comparison there were no taxa identified that are considered indicators of fecal contamination, therefore the data was reanalyzed to include only the nearshore samples. A comparison focusing on just the nearshore sites including GCM (river mouth) indicates GCM has more taxa that differ significantly with LDA effect sizes greater than 3.0 at the lowest level of classification (Figure 5B; taxa LDA >2.0 Supplementary Table S4). Most of the differing taxa in GCM samples belonged to the phylum Proteobacteria whereas in the JP samples they were mainly in the Bacteroidetes and a mixture of Proteobacteria and Actinobacteria in the site 63rd and WH samples. Bacteroidetes are a common phylum in fecal samples but since the taxa that differed are not classified to the genus level it is not possible to speculate if they are feces associated. However, at LDA 2.0 unclassified Enterobacteriaceae are significant in the JP sample (Supplementary Table S4). This group associated with fecal contamination. Of the nearshore site JP had the highest frequency of elevated *E. coli* MPN values (Supplementary Table S5). This indicates that targeted metagenomic analysis can provide additional data of fecal contamination.

### Microbial Community and *E. coli*

Although the incidences of elevated *E. coli* MPN values were limited, correlation analysis indicated that there were positive correlations with other biomarkers and bacterial genera commonly reported from fecal samples. There were few samples that had elevated *E. coli* (>235 CFU/MPN per 100 ml) that would lead to closures of recreational waters (Supplementary Table S5; U.S. EPA, 2012). Spearman Rho analysis of bacterial taxa relative abundances and *E. coli* MPN (sorted into bins) indicated there were 52 genera with significant positive correlations and 1 negative correlation (Supplementary Table S6). Notable is the positive correlation with several genera in the phylum Firmicutes that are associated with fecal contamination, *Enterococcus*, *Blautia*, *Faecalibacterium*, *Clostridium*, *Collinsella*, and *Selenomonas* as well as *Bacteroides* from the phylum Bacteroidetes.

*Enterococcus* (Wheeler et al., 2002) and *Bacteroides* (Bernhard and Field, 2000) are often used as fecal indicator species, and more recently, *Blautia* (Koskey et al., 2014; Eren et al., 2015) and *Dialister* (Jeong et al., 2011) have also been proposed as indicator species. A species within *Clostridium* (*C. perfringens*) has been suggested as a reliable indicator of water quality in tropical areas (Fujioka and Shizumura, 1985; Fujioka et al., 1997), where traditional indicators such as *E. coli*, and enterococci, are commonly found in the environment (Byappanahalli and Ishii, 2011). *Collinsella*, *Blautia*, and *Faecalibacterium*, are examples of commonly found members of gut microbiomes that correlated with *E. coli* MPNs. With the availability of high throughput sequencing technology, others have suggested that community analysis may be an additional means to assess water quality and can be applied to microbial source tracking (Newton et al., 2013; Henry et al., 2016).

**FIGURE 6:**
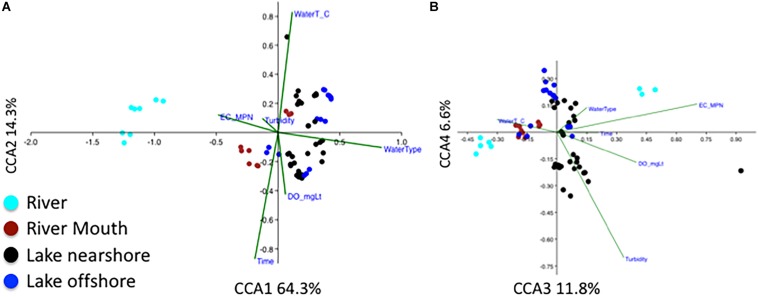
Canonical correspondence analysis (CCA) of relative abundance of bacterial genera, water type (river, river mouth, nearshore, and offshore), *Escherichia coli* most probable number (ECMPN), and the environmental variables water temperature (WaterT_C), water turbidity (Turbidity_FNU), dissolved oxygen in mgL^– 1^ (DOmgLt) and sampling date (time). **(A)** CCA1 and 2 explain 64.3 and 14.4% and **(B)** CCA3 and CCA4 explain 11.8 and 6.6% of the total constrained variation, respectively. Number of samples *n* = 63, overall *P* = 0.001.

The influence of physical conditions on overall microbial community was also examined (Figure 6) using CCA. Factors corresponding to the differences in the bacterial communities were water type (river water being most distant from lake) along the first axis explaining 64.3% of the variation; sampling time, water temperatures and dissolved oxygen along the second axis (14.3%) (Figure 6A); and *E. coli* MPN and turbidity along the third and fourth axes (Figure 6B) (100 permutations, *p* < 0.01). The influence of water type describing the microbial community is like the finding of beta diversity analysis due to differences of the river community to the other water types (Figure 3A). A secondary factor was the difference in communities with sampling time (Figure 3B) that was shown by CCA to be influenced by increasing dissolved oxygen and decreasing water temperatures (Figures 6A,B). The *E. coli* MPN corresponded to the three river samples collected on the third sampling date after rainfall (Figure 6B). This suggests that both shoreline sources (e.g., gulls, runoff, nearshore sand-water interactions) and large-scale processes (e.g., waves, currents, lake turnover), as well as time of year, are likely to influence changes in community along the shoreline. The relative impact of these factors could have inter-annual variation. Integration of physical modeling and multiple years of data could help resolve some of these interactions.

### Implications for Mitigation Strategies

After the recognition that aquatic systems have become degraded, it is essential to develop strategies to restore compromised ecosystems through appropriate remedial actions and in many instances, those actions tend to be site-specific. For instance, at the study locations, nonpoint sources of microbial contamination by shoreline birds (gulls), has been previously identified as a major contributing factor using microbial source tracking and gull deterrence activities have significantly improved shoreline water quality in recent years (Nevers et al., 2018). Similarly, the periodic presence of the human marker *Bacteroides* HF183 (Byappanahalli et al., 2015) has been a cause of concern for the potential significant human health threat; previously, the Grand Calumet River was the presumptive source of any human contamination. Results presented here, however, indicate that the river, even following a rain event, was likely not impacting shoreline bacterial communities. There may, therefore, be additional sources of human contamination currently unknown impacting these shoreline locations. At Jeorse Park and 63rd, the shoreline configuration has also been identified as contributing to the persistence of bacterial contamination due to the tendency toward accumulation and the decrease in circulation (Ge et al., 2012; Byappanahalli et al., 2015). Because the river is not the major exogenous source of microbes into the nearshore lake sites, efforts to curtail river flow toward recreational beach areas would not be a sufficient means to decrease beach closures. Efforts toward identifying and mitigating shoreline or nonpoint sources, such as decreasing gull presence (Nevers et al., 2018) and reducing other contributions (e.g., shoreline algae Whitman et al., 2014b), would likely have a greater impact on eliminating beach closures. By incorporating emerging technologies, such as the microbiome, into water quality monitoring programs will be helpful to study long-term changes and underlaying factors that influence these changes which are difficult to elucidate with traditional monitoring programs.

To summarize using a targeted 16S rRNA gene sequencing approach to analyze aquatic microbial communities demonstrated it is possible (1) to identify sources of contamination using a consortium of microbes as an index of pollutant sources rather than using one or two traditional indicators (e.g., *E. coli*, enterococci) often used in monitoring programs. Using this approach, we were able to show that the Grand Calumet River had minimal influence on shoreline water quality at the study sites, indicating that the sources contributing to high *E. coli* levels (e.g., at JP, WH) were more likely internal (e.g., shoreline birds, sand, *Cladophora*) with an intermittent contamination from GCR. (2) The data gathered from this research will be useful to management agencies, such as U.S. EPA and Indiana Department of Environmental Management, for addressing water quality restoration efforts currently under implementation at the study locations. An example will be delisting shorelines as impaired for beneficial use such as recreation. This can begin the process to modify acceptable approaches by government agencies to identify microbially contaminated locations to implement effective mitigation.

## Author Contributions

All authors wrote and approved the final version of the manuscript. MN and MB conceived the project. MB, MN, and CN performed the sample designing, contributed to methodology, and wrote the manuscript. CN analyzed the data.

## Conflict of Interest Statement

The authors declare that the research was conducted in the absence of any commercial or financial relationships that could be construed as a potential conflict of interest.

## Footnotes

1 http://folk.uio.no/ohammer/past/index.html

2 http://www.hammondsd.com/cso-discharge-activity-maps/

## Supplementary Material

FIGURE S1 Rarefaction curves of alpha diversity measures **(A)** Shannon Diversity and **(B)** PD whole tree (Faith’s Phylogenetic Diversity). Each line represents a single sample.

TABLE S1 Relative proportion of phyla represented in Lake Michigan water sample.

TABLE S2 LEfSe results of a comparison of different Lake Michigan water sources, taxa at all levels with LDA > 2.0 are listed.

TABLE S3 LEfSe results of a comparison of Lake Michigan sampling sites, taxa at all levels with LDA > 2.0 are listed GCR.

TABLE S4 LEfSe results of a comparison of only the nearshore Lake Michigan sampling sites, taxa at all levels with LDA > 2.0 are listed.

TABLE S5 Environmental conditions and *E. coli* most probable numbers (MPN) in triplicate samples from each sampling location on three collection dates.

TABLE S6 Significant Spearman rho correlations *E. coli* most probable numbers (MPN) binned and relative abundances of bacteria at genus level.
