# Supplementary figures and images for: Bacterial Community 16S rRNA Gene Sequencing Characterizes Riverine Microbial Impact on Lake Michigan
Source: Front Microbiol. 2019 May 14;10:996. doi: 10.3389/fmicb.2019.00996 (PMC6527805; doi:10.3389/fmicb.2019.00996)

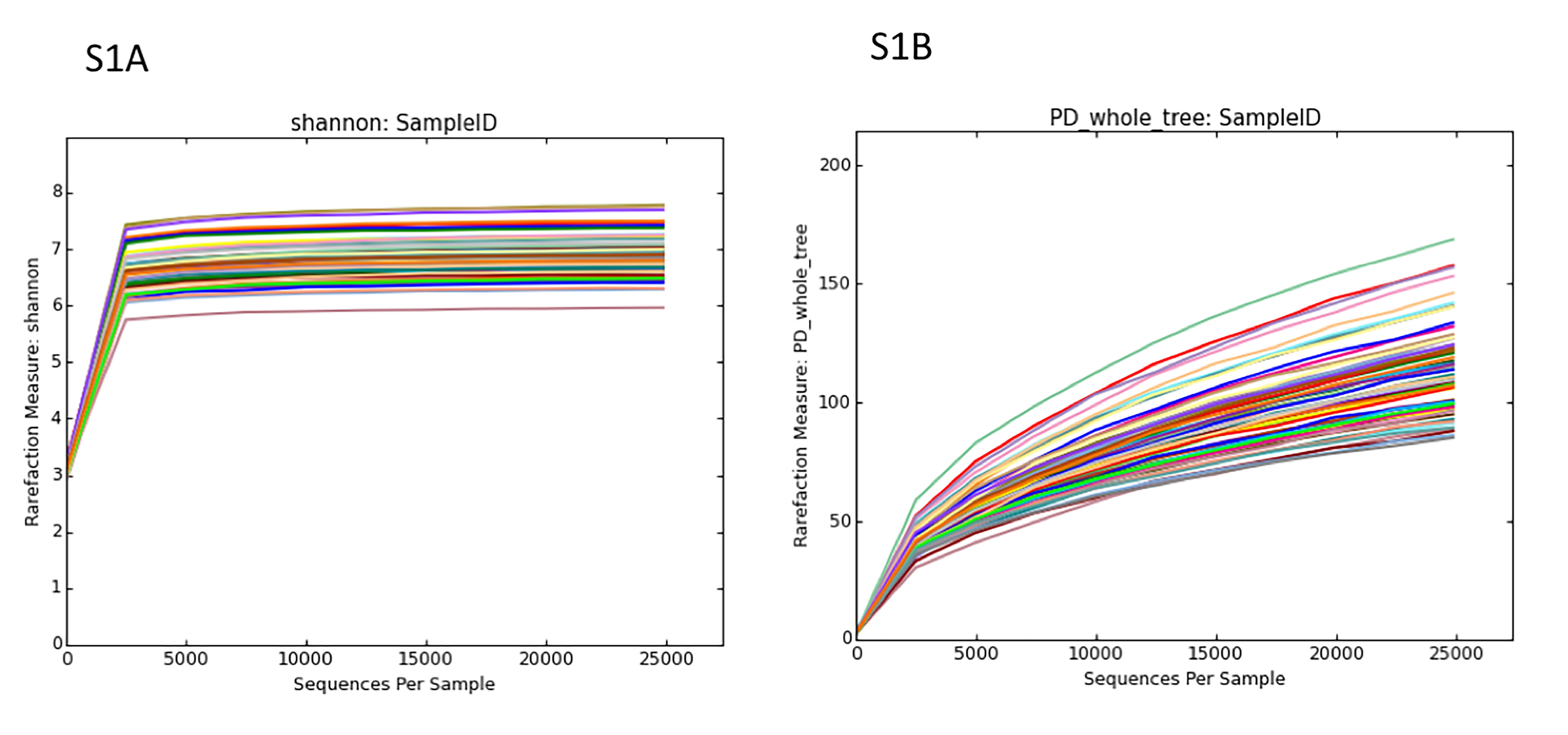

Supplement: FIGURE S1 — Rarefaction curves of alpha diversity measures (A) Shannon Diversity and (B) PD whole tree (Faith’s Phylogenetic Diversity). Each line represents a single sample. [file Image_1.jpg]
