# Supplementary material for: Bacterial Community 16S rRNA Gene Sequencing Characterizes Riverine Microbial Impact on Lake Michigan
Source: Front Microbiol. 2019 May 14;10:996. doi: 10.3389/fmicb.2019.00996 (PMC6527805; doi:10.3389/fmicb.2019.00996)
Supplement: TABLE S1 — Relative proportion of phyla represented in Lake Michigan water sample. [file Data_Sheet_1.pdf]

Tables S1: Relative proportion of phyla represented in Lake Michigan water sample.

Phylum	**Mean**	Stand. dev	Min	Max
Unclassified Bacteria	**0.93%**	0.41%	0.33%	2.23%
AC1	**0.00%**	0.00%	0.00%	0.01%
AD3	**0.00%**	0.00%	0.00%	0.00%
Acidobacteria	**0.06%**	0.07%	0.00%	0.25%
Actinobacteria	**26.77%**	8.91%	3.45%	42.05%
Armatimonadetes	**0.01%**	0.01%	0.00%	0.08%
BHI80-139	**0.00%**	0.00%	0.00%	0.01%
Bacteroidetes	**22.75%**	6.15%	10.07%	36.26%
Caldiserica	**0.00%**	0.00%	0.00%	0.00%
Caldithrix	**0.00%**	0.00%	0.00%	0.01%
Chlamydiae	**0.00%**	0.00%	0.00%	0.01%
Chlorobi	**0.08%**	0.08%	0.00%	0.31%
Chloroflexi	**0.93%**	0.86%	0.02%	3.60%
Cyanobacteria	**1.51%**	1.33%	0.03%	5.71%
Deferribacteres	**0.00%**	0.00%	0.00%	0.00%
Elusimicrobia	**0.02%**	0.02%	0.00%	0.10%
FCPU426	**0.00%**	0.00%	0.00%	0.01%
Fibrobacteres	**0.01%**	0.01%	0.00%	0.05%
Firmicutes	**0.34%**	0.28%	0.01%	1.81%
Fusobacteria	**0.01%**	0.01%	0.00%	0.06%
GAL15	**0.00%**	0.00%	0.00%	0.00%
GN02	**0.01%**	0.01%	0.00%	0.05%
GN04	**0.00%**	0.00%	0.00%	0.02%
Gemmatimonadetes	**0.03%**	0.03%	0.00%	0.11%
H-178	**0.00%**	0.00%	0.00%	0.01%
Kazan-3B-28	**0.00%**	0.00%	0.00%	0.00%
Lentisphaerae	**0.00%**	0.00%	0.00%	0.00%
MVS-104	**0.00%**	0.00%	0.00%	0.00%
NC10	**0.00%**	0.00%	0.00%	0.01%
Nitrospirae	**0.04%**	0.04%	0.00%	0.16%
OC31	**0.00%**	0.00%	0.00%	0.01%
OD1	**0.00%**	0.01%	0.00%	0.02%
OP3	**0.00%**	0.00%	0.00%	0.01%
OP8	**0.00%**	0.00%	0.00%	0.01%
Planctomycetes	**0.02%**	0.02%	0.00%	0.07%
Proteobacteria	**45.65%**	5.87%	35.43%	61.62%
SR1	**0.01%**	0.02%	0.00%	0.08%

Spirochaetes	**0.03%**	0.02%	0.00%	0.16%
TM6	**0.01%**	0.01%	0.00%	0.06%
TM7	**0.06%**	0.07%	0.00%	0.37%
Tenericutes	**0.00%**	0.01%	0.00%	0.05%
Verrucomicrobia	**0.69%**	0.57%	0.01%	2.18%
WPS-2	**0.00%**	0.00%	0.00%	0.02%
WS2	**0.00%**	0.00%	0.00%	0.00%
WS3	**0.01%**	0.01%	0.00%	0.04%
WS5	**0.00%**	0.01%	0.00%	0.09%
WS6	**0.00%**	0.00%	0.00%	0.00%
WWE1	**0.00%**	0.00%	0.00%	0.00%
ZB3	**0.01%**	0.01%	0.00%	0.08%
[Thermi]	**0.00%**	0.00%	0.00%	0.01%

Supplemental Table 2: LEfSe results of a comparison of different Lake Michigan water sources, taxa at all levels with LDA >2.0 are listed

River

**Taxon**	**value**	**LDA**	**P**
Acidobacteria.c_Solibacteres.o_Solibacterales_Other	1.18	3.29	0.034148
Actinobacteria.c_Actinobacteria.o_Actinomycetales.f_Microbacteriaceae	3.98	3.61	0.000000
Actinobacteria.c_Actinobacteria.o_Actinomycetales.f_Microbacteriaceae.g_CandidatusAquiluna	3.97	3.60	0.000000
Actinobacteria.c_Coriobacteriia.o_Coriobacteriales.f_Coriobacteriaceae.g_Collinsella	1.00	3.03	0.004184
Bacteroidetes	5.53	4.85	0.000063
Bacteroidetes.c_Bacteroidia	3.45	3.00	0.000227
Bacteroidetes.c_Bacteroidia.o_Bacteroidales	3.39	2.92	0.001764
Bacteroidetes.c_Bacteroidia.o_Bacteroidales_unclassified	2.49	2.19	0.000000
Bacteroidetes.c__Bacteroidia.o__Bacteroidales.f___Paraprevotellaceae_.g_YRC22	1.18	2.33	0.000157
Bacteroidetes.c_Bacteroidia.o_Bacteroidales.f_Bacteroidaceae	2.76	2.46	0.000370
Bacteroidetes.c_Bacteroidia.o_Bacteroidales.f_Bacteroidaceae.g_Bacteroides	2.76	2.46	0.000370
Bacteroidetes.c_Bacteroidia.o_Bacteroidales.f_Porphyromonadaceae	2.61	2.32	0.000000
Bacteroidetes.c_Bacteroidia.o_Bacteroidales.f_Porphyromonadaceae.g_Paludibacter	2.61	2.31	0.000000
Bacteroidetes.c_Bacteroidia.o_Bacteroidales.f_Prevotellaceae	2.57	2.30	0.000000
Bacteroidetes.c_Bacteroidia.o_Bacteroidales.f_Prevotellaceae.g_Prevotella	2.57	2.30	0.000000
Bacteroidetes.c_Bacteroidia.o_Bacteroidales.f_Rikenellaceae_unclassified	1.18	2.29	0.004184
Bacteroidetes.c_Cytophagia	5.05	4.49	0.000072
Bacteroidetes.c_Cytophagia.o_Cytophagales	5.05	4.50	0.000073
Bacteroidetes.c_Flavobacteriia	5.24	4.72	0.000089
Bacteroidetes.c_Flavobacteriia.o_Flavobacteriales	5.24	4.72	0.000089
Bacteroidetes.c__Flavobacteriia.o__Flavobacteriales.f___Weeksellaceae_	2.47	2.20	0.035534
Bacteroidetes.c__Flavobacteriia.o__Flavobacteriales.f___Weeksellaceae_.g_Cloacibacterium	2.45	2.20	0.002812
Bacteroidetes.c_Flavobacteriia.o_Flavobacteriales.f_Cryomorphaceae	4.67	4.18	0.000017
Bacteroidetes.c_Flavobacteriia.o_Flavobacteriales.f_Cryomorphaceae.g_Fluviicola	4.67	4.18	0.000017

Bacteroidetes.c_Flavobacteriia.o_Flavobacteriales.f_Cryomorphaceae.g_Wandonia	1.18	2.44	0.020955
Bacteroidetes.c_Flavobacteriia.o_Flavobacteriales.f_Flavobacteriaceae	5.07	4.60	0.000112
Bacteroidetes.c_Flavobacteriia.o_Flavobacteriales.f_Flavobacteriaceae.g_Flavobacterium	5.07	4.60	0.000112
Bacteroidetes.c_Sphingobacteriia.o_Sphingobacteriales.f_Sphingobacteriaceae.g_Sphingobacterium	1.00	2.31	0.004184
Chloroflexi.c_Dehalococcoidetes.o_Dehalococcoidales_unclassified	1.00	2.02	0.020955
Fibrobacteres.c_Fibrobacteria.o_Fibrobacterales_unclassified	1.48	2.10	0.019583
Firmicutes	3.67	3.07	0.026888
Firmicutes.c_Clostridia	3.57	2.93	0.032200
Firmicutes.c_Clostridia.o_Clostridiales	3.36	2.84	0.000292
Firmicutes.c_Clostridia.o_Clostridiales.f_Clostridiaceae	2.65	2.20	0.002113
Firmicutes.c_Clostridia.o_Clostridiales.f_Clostridiaceae.g_Clostridium	2.62	2.19	0.003480
Firmicutes.c_Clostridia.o_Clostridiales.f_Lachnospiraceae_unclassified	2.75	2.10	0.038727
Firmicutes.c_Clostridia.o_Clostridiales.f_Lachnospiraceae.g_Blautia	1.30	2.13	0.004184
Firmicutes.c_Clostridia.o_Clostridiales.f_Syntrophomonadaceae	1.00	2.23	0.004184
Firmicutes.c_Clostridia.o_Clostridiales.f_Syntrophomonadaceae.g_Syntrophomonas	1.00	2.55	0.004184
Firmicutes.c_Clostridia.o_Clostridiales.f_Veillonellaceae.g_Selenomonas	1.00	2.56	0.004184
Firmicutes.c_Clostridia.o_Clostridiales.f_Veillonellaceae.g_vadinHB04	1.18	2.13	0.001873
Fusobacteria	2.40	2.06	0.000001
Fusobacteria.c_Fusobacteriia	2.40	2.06	0.000001
H_178_unclassified	1.54	2.46	0.000000
Kazan_3B_28_unclassified	1.40	2.62	0.000000
Nitrospirae	2.88	2.55	0.000000
Nitrospirae.c_Nitrospira	2.88	2.55	0.000000
Nitrospirae.c_Nitrospira.o_Nitrospirales	2.88	2.55	0.000000
Nitrospirae.c_Nitrospira.o_Nitrospirales.f_Nitrospiraceae	2.86	2.53	0.000000
Nitrospirae.c_Nitrospira.o_Nitrospirales.f_Nitrospiraceae.g_Nitrospira	2.86	2.53	0.000000
Proteobacteria	5.75	4.88	0.000000
Proteobacteria.c_Alphaproteobacteria.o_Rhizobiales	4.17	3.78	0.000000
Proteobacteria.c_Alphaproteobacteria.o_Rhizobiales.f_Hyphomicrobiaceae	2.44	2.02	0.000003

Proteobacteria.c_Alphaproteobacteria.o_Rhizobiales.f_Rhizobiaceae	3.12	2.77	0.000015
Proteobacteria.c_Alphaproteobacteria.o_Rhizobiales.f_Rhizobiaceae_unclassified	4.10	3.76	0.000007
Proteobacteria.c_Alphaproteobacteria.o__Rhizobiales.f_Rhizobiaceae.g_Agrobacterium	3.12	2.77	0.000011
Proteobacteria.c_Alphaproteobacteria.o_Rhodospirillales.f_Rhodospirillaceae	2.45	2.10	0.012934
Proteobacteria.c_Alphaproteobacteria.o_Rhodospirillales.f_Rhodospirillaceae.g__Azospirillum	2.37	2.08	0.000003
Proteobacteria.c_Alphaproteobacteria.o_Sphingomonadales_Other	2.67	2.31	0.002503
Proteobacteria.c_Alphaproteobacteria.o_Sphingomonadales_unclassified	4.82	4.35	0.000003
Proteobacteria.c_Alphaproteobacteria.o_Sphingomonadales.f_Erythrobacteraceae_unclassified	3.95	3.65	0.000000
Proteobacteria.c_Alphaproteobacteria.o_Sphingomonadales.f_Sphingomonadaceae	3.94	3.61	0.000000
Proteobacteria.c_Alphaproteobacteria.o_Sphingomonadales.f_Sphingomonadaceae.g_Kaistobacter	2.61	2.34	0.000030
Proteobacteria.c_Alphaproteobacteria.o_Sphingomonadales.f_Sphingomonadaceae.g_Novosphingobium	3.91	3.59	0.000000
Proteobacteria.c_Betaproteobacteria	5.60	4.80	0.000003
Proteobacteria.c_Betaproteobacteria.o_Burkholderiales	5.56	4.80	0.000045
Proteobacteria.c_Betaproteobacteria.o_Burkholderiales.f_Comamonadaceae_Other	5.20	4.86	0.000000
Proteobacteria.c_Betaproteobacteria.o_Burkholderiales.f_Comamonadaceae_unclassified	5.05	4.55	0.000006
Proteobacteria.c_Betaproteobacteria.o_Burkholderiales.f_Comamonadaceae.g_Comamonas	2.32	2.01	0.000080
Proteobacteria.c_Betaproteobacteria.o_Burkholderiales.f_Comamonadaceae.g_Hydrogenophaga	3.65	3.25	0.000000
Proteobacteria.c_Betaproteobacteria.o_Burkholderiales.f_Comamonadaceae.g_Tepidimonas	2.75	2.51	0.000657
Proteobacteria.c_Betaproteobacteria.o_Burkholderiales.f_Comamonadaceae.g_Variovorax	2.40	2.09	0.000008
Proteobacteria.c_Betaproteobacteria.o_Burkholderiales.f_Oxalobacteraceae	4.53	4.22	0.000000
Proteobacteria.c_Betaproteobacteria.o_Burkholderiales.f_Oxalobacteraceae.g_Polynucleobacter	4.53	4.22	0.000000
Proteobacteria.c_Betaproteobacteria.o_Rhodocyclales.f_Rhodocyclaceae	3.52	3.21	0.000000
Proteobacteria.c_Betaproteobacteria.o_Rhodocyclales.f_Rhodocyclaceae.g_C39	3.34	3.05	0.000000
Proteobacteria.c_Betaproteobacteria.o_Rhodocyclales.f_Rhodocyclaceae.g_Dechloromonas	2.83	2.49	0.000001
Proteobacteria.c_Betaproteobacteria.o_Rhodocyclales.f_Rhodocyclaceae.g_Sterolibacterium	1.18	2.61	0.000156
Proteobacteria.c_Epsilonproteobacteria	4.04	3.76	0.000018
Proteobacteria.c_Epsilonproteobacteria.o_Campylobacterales	4.04	3.76	0.000018
Proteobacteria.c_Epsilonproteobacteria.o_Campylobacterales.f_Campylobacteraceae	4.01	3.72	0.000019
Proteobacteria.c_Epsilonproteobacteria.o_Campylobacterales.f_Campylobacteraceae_unclassified	1.18	2.18	0.001292

Proteobacteria.c_Epsilonproteobacteria.o_Campylobacterales.f_Campylobacteraceae.g_Arcobacter	4.01	3.72	0.000021
Proteobacteria.c_Epsilonproteobacteria.o_Campylobacterales.f_Helicobacteraceae	2.93	2.62	0.000012
Proteobacteria.c_Epsilonproteobacteria.o_Campylobacterales.f_Helicobacteraceae_unclassified	1.40	2.50	0.039115
Proteobacteria.c_Epsilonproteobacteria.o_Campylobacterales.f_Helicobacteraceae.g_Sulfuricurvum	2.88	2.56	0.000021
Proteobacteria.c_Gammaproteobacteria.o_Aeromonadales	3.18	2.73	0.000156
Proteobacteria.c_Gammaproteobacteria.o_Alteromonadales.f_Alteromonadaceae	2.76	2.45	0.000003
Proteobacteria.c_Gammaproteobacteria.o_Alteromonadales.f_Alteromonadaceae.g_Cellvibrio	2.74	2.43	0.000002
Proteobacteria.c_Gammaproteobacteria.o_Alteromonadales.f_HTCC2188_unclassified	2.50	2.18	0.000000
Proteobacteria.c_Gammaproteobacteria.o_Pseudomonadales.f_Moraxellaceae	3.37	3.03	0.000032
Proteobacteria.c_Gammaproteobacteria.o_Pseudomonadales.f_Moraxellaceae_unclassified	2.37	2.02	0.000025
Proteobacteria.c_Gammaproteobacteria.o_Pseudomonadales.f_Moraxellaceae.g_Enhydrobacter	2.40	2.04	0.000685
Proteobacteria.c_Gammaproteobacteria.o_Pseudomonadales.f_Moraxellaceae.g_Perlucidibaca	2.43	2.12	0.000000
SR1_unclassified	2.68	2.37	0.000000
WWE1.c___Cloacamonae_.o___Cloacamonales_.f_CW_1_unclassified	1.18	2.30	0.000157

River mouth

Taxon	value	LDA	P
Actinobacteria.c_Acidimicrobiia	4.47	4.1	0.0000
Actinobacteria.c_Acidimicrobiia.o_Acidimicrobiales	4.42	4.1	0.0000
Actinobacteria.c_Acidimicrobiia.o_Acidimicrobiales_unclassified	3.45	3.1	0.0000
Actinobacteria.c_Acidimicrobiia.o_Acidimicrobiales.f_C111_unclassified	4.42	4.1	0.0000
Actinobacteria.c_Actinobacteria.o_Actinomycetales.f_Micrococcaceae_unclassified	0.95	3.6	0.0092
Actinobacteria.c_Thermoleophilia	3.09	2.7	0.0001
Actinobacteria.c_Thermoleophilia.o_Gaiellales_unclassified	3.08	2.8	0.0000
Bacteroidetes.c_Sphingobacteriia	4.58	4.2	0.0000
Bacteroidetes.c_Sphingobacteriia.o_Sphingobacteriales_unclassified	4.57	4.2	0.0001
Chlorobi	3.38	3.0	0.0000
Chlorobi.c_OPB56_unclassified	3.38	3.0	0.0000
Firmicutes.c_Bacilli	3.34	3.0	0.0052

Firmicutes.c_Bacilli.o_Bacillales	3.27	3.0	0.0032
Firmicutes.c_Bacilli.o_Bacillales.f_Bacillaceae	3.27	3.0	0.0001
Firmicutes.c_Bacilli.o_Bacillales.f_Bacillaceae.g_Bacillus	3.27	3.0	0.0001
Gemmatimonadetes	2.59	2.1	0.0002
Gemmatimonadetes.c_Gemmatimonadetes	2.57	2.1	0.0007
Gemmatimonadetes.c_Gemmatimonadetes.o_Gemmatimonadales	2.56	2.1	0.0001
Gemmatimonadetes.c_Gemmatimonadetes.o_Gemmatimonadales.f_Gemmatimonadaceae	2.56	2.2	0.0001
Gemmatimonadetes.c_Gemmatimonadetes.o_Gemmatimonadales.f_Gemmatimonadaceae.g_Gemmatimonas	2.56	2.2	0.0001
Proteobacteria.c_Alphaproteobacteria.o_Rhizobiales_unclassified	4.04	3.7	0.0000
Proteobacteria.c_Alphaproteobacteria.o_Rhizobiales.f_Methylocystaceae	3.25	2.9	0.0000
Proteobacteria.c_Alphaproteobacteria.o_Rhizobiales.f_Methylocystaceae.g_Methylosinus	3.25	2.9	0.0000
Proteobacteria.c_Alphaproteobacteria.o_Rhodobacterales	4.54	3.9	0.0002
Proteobacteria.c_Alphaproteobacteria.o_Rhodobacterales.f_Rhodobacteraceae	4.54	3.9	0.0001
Proteobacteria.c_Alphaproteobacteria.o__Rhodobacterales.f_Rhodobacteraceae_unclassified	2.43	2.1	0.0000
Proteobacteria.c_Alphaproteobacteria.o_Rhodobacterales.f_Rhodobacteraceae.g_Rhodobacter	4.54	3.9	0.0001
Proteobacteria.c_Alphaproteobacteria.o_Rhodospirillales.f_Rhodospirillaceae_unclassified	2.81	2.2	0.0001
Proteobacteria.c_Alphaproteobacteria.o_Sphingomonadales.f_Sphingomonadaceae_Other	3.49	3.2	0.0000
Proteobacteria.c_Betaproteobacteria_unclassified	3.75	3.4	0.0000
Proteobacteria.c_Betaproteobacteria.o_Burkholderiales_unclassified	4.28	3.8	0.0000
Proteobacteria.c_Betaproteobacteria.o_Burkholderiales.f_Comamonadaceae.g_Roseateles	2.40	2.0	0.0000
Proteobacteria.c_Betaproteobacteria.o_Burkholderiales.f_Oxalobacteraceae_unclassified	3.64	3.2	0.0000
Proteobacteria.c_Betaproteobacteria.o_Rhodocyclales.f_Rhodocyclaceae_unclassified	3.85	3.3	0.0401
Proteobacteria.c_Deltaproteobacteria.o_Bdellovibrionales.f_Bacteriovoracaceae_unclassified	3.00	2.4	0.0421
Proteobacteria.c_Gammaproteobacteria	4.50	4.1	0.0000
Proteobacteria.c_Gammaproteobacteria.o_Alteromonadales	4.09	3.7	0.0000
Proteobacteria.c_Gammaproteobacteria.o_Alteromonadales.f_125ds10_unclassified	3.16	2.8	0.0000
Proteobacteria.c_Gammaproteobacteria.o_Alteromonadales.f_HTCC2188	4.01	3.7	0.0000
Proteobacteria.c_Gammaproteobacteria.o_Alteromonadales.f_HTCC2188.g_HTCC	4.01	3.7	0.0000
Proteobacteria.c_Gammaproteobacteria.o_Chromatiales	3.73	3.5	0.0000

Proteobacteria.c_Gammaproteobacteria.o_Chromatiales_unclassified	3.13	2.8	0.0001
Proteobacteria.c_Gammaproteobacteria.o_Chromatiales.f_Halothiobacillaceae	3.72	3.5	0.0000
Proteobacteria.c_Gammaproteobacteria.o_Chromatiales.f_Halothiobacillaceae.g_Thiovirga	3.72	3.5	0.0000
Proteobacteria.c_Gammaproteobacteria.o_Methylococcales	3.19	2.9	0.0000
Proteobacteria.c_Gammaproteobacteria.o_Methylococcales.f_Methylococcaceae	3.18	2.9	0.0000
Proteobacteria.c_Gammaproteobacteria.o_Methylococcales.f_Methylococcaceae.g_Methylocaldum	3.17	2.9	0.0000
Proteobacteria.c_Gammaproteobacteria.o_Pseudomonadales	3.56	3.2	0.0000
Proteobacteria.c_Gammaproteobacteria.o_Pseudomonadales.f_Pseudomonadaceae	3.47	3.1	0.0000
Proteobacteria.c_Gammaproteobacteria.o_Pseudomonadales.f_Pseudomonadaceae.g_Pseudomonas	3.47	3.1	0.0000
Proteobacteria.c_Gammaproteobacteria.o_Xanthomonadales	3.73	3.3	0.0001
Proteobacteria.c_Gammaproteobacteria.o_Xanthomonadales.f_Sinobacteraceae_unclassified	3.70	3.3	0.0000
Proteobacteria.c_TA18	3.48	3.2	0.0004
Proteobacteria.c_TA18.o_PHOS_HD29_unclassified	3.48	3.2	0.0004
Verrucomicrobia.c___Pedosphaerae_	3.28	3.0	0.0000
Verrucomicrobia.c___Pedosphaerae_.o___Pedosphaerales_	3.28	3.0	0.0000
Verrucomicrobia.c___Pedosphaerae_.o___Pedosphaerales_.f_R4_41B_unclassified	3.28	3.0	0.0000
Verrucomicrobia.c___Spartobacteria_.o___Chthoniobacterales_.f___Chthoniobacteraceae_unclassified	2.63	2.3	0.0000
Verrucomicrobia.c_Verrucomicrobiae	2.84	2.5	0.0000
Verrucomicrobia.c_Verrucomicrobiae.o_Verrucomicrobiales	2.84	2.5	0.0000
Verrucomicrobia.c_Verrucomicrobiae.o_Verrucomicrobiales.f_Verrucomicrobiaceae	2.67	2.3	0.0001
Verrucomicrobia.c_Verrucomicrobiae.o_Verrucomicrobiales.f_Verrucomicrobiaceae_unclassified	2.34	2.0	0.0000
Verrucomicrobia.c_Verrucomicrobiae.o_Verrucomicrobiales.f_Verrucomicrobiaceae.g_Prosthecobacter	2.60	2.3	0.0000

Lake nearshore

Taxon	value	LDA	P
Acidobacteria	3.00	2.58	0.0000027
Acidobacteria.c_Acidobacteria_6	2.63	2.24	0.0000014
Acidobacteria.c_Holophagae	2.33	2.02	0.0000004
Acidobacteria.c_Holophagae.o_Holophagales	2.33	2.02	0.0000004

Acidobacteria.c_Holophagae.o_Holophagales.f_Holophagaceae_unclassified	2.32	2.01	0.0000004
Actinobacteria	5.49	5.07	0.0000056
Actinobacteria.c_Actinobacteria	5.47	5.04	0.0000015
Actinobacteria.c_Actinobacteria.o_Actinomycetales	5.44	5.01	0.0000020
Actinobacteria.c_Actinobacteria.o_Actinomycetales.f_ACK_M1_unclassified	5.36	4.96	0.0000047
Actinobacteria.c_Actinobacteria.o_Actinomycetales.f_Microbacteriaceae_unclassified	4.50	4.15	0.0000005
Bacteroidetes.c_Bacteroidia.o_Bacteroidales.f_ML635J_40_unclassified	2.88	2.58	0.0195284
Chloroflexi	4.09	3.78	0.0000838
Chloroflexi.c_Ellin6529_unclassified	2.40	2.03	0.0000001
Chloroflexi.c_SL56_unclassified	4.07	3.77	0.0000888
Cyanobacteria.c_Chloroplast.o_Cryptophyta_unclassified	2.55	2.32	0.0165261
Elusimicrobia	2.48	2.13	0.0000000
Elusimicrobia.c_Elusimicrobia	2.42	2.07	0.0000001
Firmicutes.c__Clostridia.o__Clostridiales.f___Acidaminobacteraceae_	2.46	2.09	0.0000016
Nitrospirae.c__Nitrospira.o__Nitrospirales.f___Thermodesulfovibrionaceae_	2.42	2.10	0.0000026
Proteobacteria.c_Alphaproteobacteria_unclassified	3.22	2.83	0.0000000
Proteobacteria.c_Alphaproteobacteria.o_BD7_3_unclassified	2.90	2.53	0.0000000
Proteobacteria.c_Alphaproteobacteria.o_Caulobacterales.f_Caulobacteraceae	2.68	2.17	0.0014244
Proteobacteria.c_Alphaproteobacteria.o_Caulobacterales.f_Caulobacteraceae.g_Mycoplana	2.60	2.27	0.0000016
Proteobacteria.c_Alphaproteobacteria.o_Rhodobacterales.f_Hyphomonadaceae_unclassified	2.70	2.30	0.0000007
Proteobacteria.c_Alphaproteobacteria.o_Rickettsiales	3.79	3.47	0.0000003
Proteobacteria.c_Alphaproteobacteria.o_Rickettsiales.f_Pelagibacteraceae_unclassified	3.48	3.17	0.0000023
Proteobacteria.c_Alphaproteobacteria.o_Rickettsiales.f_Rickettsiaceae_unclassified	3.48	3.17	0.0000172
Proteobacteria.c_Betaproteobacteria.o_Burkholderiales.f_Burkholderiaceae_unclassified	2.36	2.00	0.0380222
Proteobacteria.c_Betaproteobacteria.o_Burkholderiales.f_Comamonadaceae.g_Limnohabitans	5.04	4.65	0.0000034
Proteobacteria.c_Betaproteobacteria.o_Burkholderiales.f_Comamonadaceae.g_Methylibium	3.26	2.93	0.0000754
Proteobacteria.c_Betaproteobacteria.o_Burkholderiales.f_Oxalobacteraceae_Other	4.64	4.28	0.0000003
Proteobacteria.c_Betaproteobacteria.o_Ellin6067_unclassified	2.63	2.27	0.0000000
Proteobacteria.c_Betaproteobacteria.o_Neisseriales	2.76	2.38	0.0000000

Proteobacteria.c_Betaproteobacteria.o_Neisseriales.f_Neisseriaceae	2.51	2.15	0.0000002
Proteobacteria.c_Betaproteobacteria.o_Neisseriales.f_Neisseriaceae.g_Deefgea	2.34	2.05	0.0000000
Proteobacteria.c_Betaproteobacteria.o_Rhodocyclales.f_Rhodocyclaceae_Other	2.76	2.34	0.0000033
Proteobacteria.c_Betaproteobacteria.o_Rhodocyclales.f_Rhodocyclaceae.g_Sulfuritalea	2.36	2.01	0.0000009
Proteobacteria.c_Deltaproteobacteria	3.78	3.20	0.0000548
Proteobacteria.c_Deltaproteobacteria_unclassified	2.63	2.28	0.0000000
Proteobacteria.c__Deltaproteobacteria.o___Entotheonellales_unclassified	2.48	2.14	0.0000001
Proteobacteria.c_Deltaproteobacteria.o_Bdellovibrionales	3.27	2.71	0.0001231
Proteobacteria.c_Deltaproteobacteria.o_Bdellovibrionales.f_Bdellovibrionaceae	3.07	2.68	0.0000000
Proteobacteria.c_Deltaproteobacteria.o_Bdellovibrionales.f_Bdellovibrionaceae.g_Bdellovibrio	3.07	2.68	0.0000000
Proteobacteria.c_Deltaproteobacteria.o_Desulfobacterales	2.46	2.11	0.0000168
Proteobacteria.c_Deltaproteobacteria.o_Desulfuromonadales	2.45	2.09	0.0000074
Proteobacteria.c_Deltaproteobacteria.o_Spirobacillales_unclassified	3.05	2.63	0.0000000
Proteobacteria.c_Gammaproteobacteria.o_Aeromonadales.f_Aeromonadaceae_unclassified	3.14	2.64	0.0001149
Proteobacteria.c__Gammaproteobacteria.o__Alteromonadales.f___Chromatiaceae_	2.77	2.37	0.0000009
Proteobacteria.c__Gammaproteobacteria.o__Alteromonadales.f___Chromatiaceae_.g_Rheinheimera	2.77	2.37	0.0000009
Proteobacteria.c_Gammaproteobacteria.o_Legionellales_unclassified	2.40	2.03	0.0000001
Proteobacteria.c_Gammaproteobacteria.o_Xanthomonadales.f_Xanthomonadaceae_unclassified	3.16	2.74	0.0000025
Spirochaetes	2.60	2.05	0.0013984
TM7	2.93	2.58	0.0000000
TM7.c_TM7_1_unclassified	2.90	2.54	0.0000000

Lake offshore

Taxon	value	LDA	P
Actinobacteria.c_Actinobacteria.o_Actinomycetales_unclassified	4.23	3.9	0.00001
Actinobacteria.c_Actinobacteria.o_Actinomycetales.f_Mycobacteriaceae	2.94	2.5	0.00300
Actinobacteria.c_Actinobacteria.o_Actinomycetales.f_Mycobacteriaceae.g_Mycobacterium	2.94	2.5	0.00300
Bacteroidetes.c_Sphingobacteriia.o_Sphingobacteriales	3.24	2.7	0.01313
Bacteroidetes.c_Sphingobacteriia.o_Sphingobacteriales.f_Sphingobacteriaceae_unclassified	3.24	2.7	0.01393

Cyanobacteria	4.41	4.1	0.00000
Cyanobacteria.c_Chloroplast	4.39	4.1	0.00000
Cyanobacteria.c_Chloroplast.o_Stramenopiles_unclassified	4.38	4.1	0.00000
Cyanobacteria.c_Synechococcophycideae	3.13	2.7	0.00002
Cyanobacteria.c_Synechococcophycideae.o_Synechococcales	3.13	2.7	0.00002
Cyanobacteria.c_Synechococcophycideae.o_Synechococcales.f_Synechococcaceae	3.13	2.7	0.00002
Cyanobacteria.c_Synechococcophycideae.o_Synechococcales.f_Synechococcaceae.g_Synechococcus	3.13	2.7	0.00002
Planctomycetes	2.43	2.1	0.01693
Proteobacteria.c_Alphaproteobacteria.o_Caulobacterales	3.74	3.4	0.00001
Proteobacteria.c_Alphaproteobacteria.o_Caulobacterales.f_Caulobacteraceae_unclassified	3.71	3.4	0.00001
Proteobacteria.c_Alphaproteobacteria.o_Rhizobiales.f_Beijerinckiaceae_Other	3.08	2.8	0.00000
Proteobacteria.c_Alphaproteobacteria.o_Rhodospirillales	4.55	4.2	0.00005
Proteobacteria.c_Alphaproteobacteria.o_Rhodospirillales.f_Acetobacteraceae_Other	2.62	2.3	0.00000
Proteobacteria.c_Alphaproteobacteria.o_Rhodospirillales.f_Acetobacteraceae_unclassified	4.54	4.2	0.00005
Proteobacteria.c_Alphaproteobacteria.o_Rickettsiales_unclassified	3.88	3.5	0.00000
Proteobacteria.c_Betaproteobacteria_Other_Other	2.94	2.4	0.00112
Proteobacteria.c_Betaproteobacteria.o_Burkholderiales.f_Alcaligenaceae_unclassified	3.48	3.0	0.00009
Proteobacteria.c_Betaproteobacteria.o_Burkholderiales.f_Comamonadaceae	5.13	4.6	0.00000
Proteobacteria.c_Betaproteobacteria.o_Burkholderiales.f_Comamonadaceae.g_Polaromonas	3.17	2.8	0.00001
Proteobacteria.c_Betaproteobacteria.o_Burkholderiales.f_Comamonadaceae.g_Rhodoferax	4.33	3.7	0.00509
Proteobacteria.c_Betaproteobacteria.o_Methylophilales	4.29	3.8	0.00002
Proteobacteria.c_Betaproteobacteria.o_Methylophilales.f_Methylophilaceae_unclassified	4.29	3.8	0.00002
Proteobacteria.c_Betaproteobacteria.o_MWH_UniP1_unclassified	3.38	3.0	0.00000
Proteobacteria.c_Deltaproteobacteria.o_MIZ46_unclassified	3.14	2.8	0.00009
Proteobacteria.c_Deltaproteobacteria.o_Myxococcales	3.01	2.6	0.00030
Proteobacteria.c_Deltaproteobacteria.o_Myxococcales.f_OM27_unclassified	2.98	2.7	0.00005
Proteobacteria.c_Deltaproteobacteria.o_PB19_unclassified	2.42	2.1	0.00005
Proteobacteria.c_Gammaproteobacteria.o_Legionellales.f_Legionellaceae_unclassified	2.88	2.4	0.00024
Proteobacteria.c_Gammaproteobacteria.o_Xanthomonadales.f_Xanthomonadaceae.g_Stenotrophomonas	1.05	2.2	0.02016

Verrucomicrobia	4.07	3.7	0.00001
Verrucomicrobia.c___Methylacidiphilae_	3.94	3.6	0.00001
Verrucomicrobia.c___Methylacidiphilae_.o_Methylacidiphilales	3.94	3.6	0.00001
Verrucomicrobia.c___Methylacidiphilae_.o_Methylacidiphilales.f_LD19_unclassified	3.94	3.6	0.00001
Verrucomicrobia.c___Spartobacteria_	3.06	2.7	0.00000
Verrucomicrobia.c___Spartobacteria_.o___Chthoniobacterales_	3.06	2.7	0.00000
Verrucomicrobia.c___Spartobacteria_.o___Chthoniobacterales_.f___Chthoniobacteraceae_	3.03	2.7	0.00004
Verrucomicrobia.c___Spartobacteria_.o___Chthoniobacterales_.f___Chthoniobacteraceae_.g_CandidatusXiphinematobacter	3.03	2.7	0.00004

Supplemental Table 3: LEfSe results of a comparison of Lake Michigan sampling sites, taxa at all levels with LDA >2.0 are listed

GCR

**Taxon**	**value**	**LDA score**	**P**
Proteobacteria.c_Betaproteobacteria.o_Neisseriales.f_Neisseriaceae_Other	2.29	2.01	0.000024
Proteobacteria.c_Alphaproteobacteria.o_Rhizobiales.f_Hyphomicrobiaceae.g_Hyphomicrobium	2.32	2.01	0.000074
Firmicutes.c_Clostridia.o_Clostridiales.f_Ruminococcaceae.g_Ruminococcus	2.40	2.05	0.005382
Fusobacteria	2.40	2.05	0.000002
Fusobacteria.c_Fusobacteriia	2.40	2.05	0.000002
Fibrobacteres_unclassified	2.26	2.05	0.000110
Actinobacteria.c_Actinobacteria.o_Actinomycetales.f_Microbacteriaceae.g_CandidatusRhodoluna	2.32	2.06	0.000641
Bacteroidetes.c_Flavobacteriia.o_Flavobacteriales.f_Flavobacteriaceae_Other	2.33	2.06	0.005255
Proteobacteria.c_Alphaproteobacteria.o_Rhodospirillales.f_Rhodospirillaceae.g_Magnetospirillum	1.48	2.06	0.015828
Firmicutes.c_Bacilli.o_Lactobacillales.f_Streptococcaceae.g_Lactococcus	1.74	2.08	0.000001
Proteobacteria.c_Betaproteobacteria.o_Burkholderiales.f_Comamonadaceae.g_Variovorax	2.40	2.09	0.000081
Proteobacteria.c_Alphaproteobacteria.o_Rhodospirillales.f_Rhodospirillaceae.g_Azospirillum	2.37	2.09	0.000000
Firmicutes.c_Bacilli.o_Lactobacillales.f_Streptococcaceae	1.78	2.09	0.000011
Proteobacteria.c_Gammaproteobacteria.o_Pseudomonadales.f_Moraxellaceae.g_Enhydrobacter	2.40	2.10	0.003872
OD1_unclassified	1.54	2.10	0.033571
Proteobacteria.c_Betaproteobacteria.o_Burkholderiales.f_Comamonadaceae.g_Comamonas	2.32	2.12	0.000680

Proteobacteria.c_Alphaproteobacteria.o_Rhizobiales.f_Hyphomicrobiaceae	2.44	2.12	0.000021
Proteobacteria.c_Gammaproteobacteria.o_Pseudomonadales.f_Moraxellaceae_unclassified	2.37	2.13	0.000194
Proteobacteria.c_Alphaproteobacteria.o_Rhodospirillales.f_Rhodospirillaceae	2.45	2.13	0.040069
H_178_unclassified	1.54	2.13	0.000001
Bacteroidetes.c__Flavobacteriia.o__Flavobacteriales.f___Weeksellaceae_	2.47	2.18	0.039890
Bacteroidetes.c__Flavobacteriia.o__Flavobacteriales.f___Weeksellaceae_.g_Cloacibacterium	2.45	2.18	0.017540
Proteobacteria.c_Gammaproteobacteria.o_Pseudomonadales.f_Moraxellaceae.g_Perlucidibaca	2.43	2.18	0.000001
Firmicutes.c_Clostridia.o_Clostridiales.f_Clostridiaceae.g_Clostridium	2.62	2.19	0.020160
Proteobacteria.c_Gammaproteobacteria.o_Alteromonadales.f_HTCC2188_unclassified	2.50	2.25	0.000001
Firmicutes.c_Clostridia.o_Clostridiales.f_Clostridiaceae	2.65	2.27	0.012353
Bacteroidetes.c_Bacteroidia.o_Bacteroidales.f_Porphyromonadaceae.g_Paludibacter	2.61	2.31	0.000005
Bacteroidetes.c_Bacteroidia.o_Bacteroidales.f_Porphyromonadaceae	2.61	2.32	0.000001
Proteobacteria.c_Alphaproteobacteria.o_Sphingomonadales.f_Sphingomonadaceae.g_Kaistobacter	2.61	2.32	0.000257
Bacteroidetes.c_Bacteroidia.o_Bacteroidales.f_Prevotellaceae	2.57	2.32	0.000000
Bacteroidetes.c_Bacteroidia.o_Bacteroidales.f_Prevotellaceae.g_Prevotella	2.57	2.32	0.000000
Proteobacteria.c_Alphaproteobacteria.o_Sphingomonadales_Other	2.67	2.33	0.005693
Firmicutes.c_Clostridia.o_Clostridiales.f_Veillonellaceae.g_vadinHB04	1.18	2.37	0.012000
SR1_unclassified	2.68	2.40	0.000000
Proteobacteria.c_Betaproteobacteria.o_Burkholderiales.f_Comamonadaceae.g_Tepidimonas	2.75	2.46	0.003479
Proteobacteria.c_Betaproteobacteria.o_Burkholderiales.f_Comamonadaceae.g_Paucibacter	1.30	2.47	0.012010
Proteobacteria.c_Gammaproteobacteria.o_Alteromonadales.f_Alteromonadaceae.g_Cellvibrio	2.74	2.47	0.000005
Bacteroidetes.c_Bacteroidia.o_Bacteroidales.f_Bacteroidaceae.g_Bacteroides	2.76	2.48	0.001189
Bacteroidetes.c_Bacteroidia.o_Bacteroidales.f_Bacteroidaceae	2.76	2.48	0.001189
Proteobacteria.c_Gammaproteobacteria.o_Alteromonadales.f_Alteromonadaceae	2.76	2.49	0.000012
Proteobacteria.c_Betaproteobacteria.o_Rhodocyclales.f_Rhodocyclaceae.g_Dechloromonas	2.83	2.56	0.000001
Nitrospirae.c_Nitrospira.o_Nitrospirales.f_Nitrospiraceae	2.86	2.57	0.000000
Nitrospirae.c_Nitrospira.o_Nitrospirales.f_Nitrospiraceae.g_Nitrospira	2.86	2.57	0.000000
Proteobacteria.c_Epsilonproteobacteria.o_Campylobacterales.f_Helicobacteraceae.g_Sulfuricurvum	2.88	2.59	0.000137
Nitrospirae.c_Nitrospira.o_Nitrospirales	2.88	2.59	0.000000

Nitrospirae	2.88	2.60	0.000000
Nitrospirae.c_Nitrospira	2.88	2.60	0.000000
Proteobacteria.c_Epsilonproteobacteria.o_Campylobacterales.f_Helicobacteraceae	2.93	2.64	0.000085
Proteobacteria.c_Alphaproteobacteria.o_Rhizobiales.f_Rhizobiaceae.g_Agrobacterium	3.12	2.75	0.000147
Proteobacteria.c_Alphaproteobacteria.o_Rhizobiales.f_Rhizobiaceae	3.12	2.75	0.000164
Firmicutes.c_Clostridia.o_Clostridiales	3.36	2.96	0.003010
Proteobacteria.c_Betaproteobacteria.o_Rhodocyclales.f_Rhodocyclaceae.g_C39	3.34	3.05	0.000005
Proteobacteria.c_Gammaproteobacteria.o_Pseudomonadales.f_Moraxellaceae	3.37	3.06	0.000372
Proteobacteria.c_Betaproteobacteria.o_Rhodocyclales.f_Rhodocyclaceae	3.52	3.21	0.000001
Proteobacteria.c_Betaproteobacteria.o_Burkholderiales.f_Comamonadaceae.g_Hydrogenophaga	3.65	3.27	0.000002
Proteobacteria.c_Betaproteobacteria.o_Rhodocyclales	3.90	3.29	0.013614
Proteobacteria.c_Alphaproteobacteria.o_Sphingomonadales.f_Sphingomonadaceae.g_Novosphingobium	3.91	3.59	0.000001
Proteobacteria.c_Alphaproteobacteria.o_Sphingomonadales.f_Sphingomonadaceae	3.94	3.61	0.000001
Actinobacteria.c_Actinobacteria.o_Actinomycetales.f_Microbacteriaceae.g_CandidatusAquiluna	3.97	3.62	0.000000
Actinobacteria.c_Actinobacteria.o_Actinomycetales.f_Microbacteriaceae	3.98	3.63	0.000000
Proteobacteria.c_Alphaproteobacteria.o_Sphingomonadales.f_Erythrobacteraceae_unclassified	3.95	3.64	0.000000
Proteobacteria.c_Epsilonproteobacteria.o_Campylobacterales.f_Campylobacteraceae.g_Arcobacter	4.01	3.74	0.000330
Proteobacteria.c_Epsilonproteobacteria.o_Campylobacterales.f_Campylobacteraceae	4.01	3.74	0.000279
Proteobacteria.c_Alphaproteobacteria.o_Rhizobiales.f_Rhizobiaceae_unclassified	4.10	3.75	0.000009
Proteobacteria.c_Epsilonproteobacteria	4.04	3.77	0.000216
Proteobacteria.c_Epsilonproteobacteria.o_Campylobacterales	4.04	3.77	0.000216
Proteobacteria.c_Alphaproteobacteria.o_Rhizobiales	4.17	3.78	0.000000
Proteobacteria.c_Betaproteobacteria.o_Burkholderiales.f_Oxalobacteraceae.g_Polynucleobacter	4.53	4.22	0.000000
Proteobacteria.c_Betaproteobacteria.o_Burkholderiales.f_Oxalobacteraceae	4.53	4.23	0.000000
Bacteroidetes.c_Flavobacteriia.o_Flavobacteriales.f_Cryomorphaceae.g_Fluviicola	4.67	4.26	0.000163
Bacteroidetes.c_Flavobacteriia.o_Flavobacteriales.f_Cryomorphaceae	4.67	4.26	0.000163
Proteobacteria.c_Alphaproteobacteria.o_Sphingomonadales_unclassified	4.82	4.36	0.000004
Bacteroidetes.c_Cytophagia	5.05	4.52	0.000547
Bacteroidetes.c_Cytophagia.o_Cytophagales	5.05	4.52	0.000548

Proteobacteria.c_Betaproteobacteria.o_Burkholderiales.f_Comamonadaceae_unclassified	5.05	4.59	0.000005
Bacteroidetes.c_Flavobacteriia.o_Flavobacteriales.f_Flavobacteriaceae.g_Flavobacterium	5.07	4.61	0.001333
Bacteroidetes.c_Flavobacteriia.o_Flavobacteriales.f_Flavobacteriaceae	5.07	4.61	0.001333
Bacteroidetes.c_Flavobacteriia	5.24	4.74	0.000825
Bacteroidetes.c_Flavobacteriia.o_Flavobacteriales	5.24	4.74	0.000825
Bacteroidetes	5.53	4.86	0.000335
Proteobacteria.c_Betaproteobacteria.o_Burkholderiales	5.56	4.86	0.000036
Proteobacteria.c_Betaproteobacteria	5.60	4.87	0.000004
Proteobacteria.c_Betaproteobacteria.o_Burkholderiales.f_Comamonadaceae_Other	5.20	4.88	0.000000
Proteobacteria	5.75	4.90	0.000001

GCM

Taxon	value	LDA score	P
Proteobacteria.c_Alphaproteobacteria.o_Ellin329_unclassified	1.65	2.03	0.025539
Proteobacteria.c_Gammaproteobacteria.o_34P16_unclassified	2.24	2.04	0.000004
Armatimonadetes.c_Armatimonadia	1.12	2.04	0.027012
Armatimonadetes.c_Armatimonadia.o_Armatimonadales	1.12	2.06	0.027012
Proteobacteria.c_Betaproteobacteria.o_Burkholderiales.f_Comamonadaceae.g_Roseateles	2.40	2.07	0.000066
Verrucomicrobia.c_Verrucomicrobiae.o_Verrucomicrobiales.f_Verrucomicrobiaceae_unclassified	2.34	2.10	0.000123
Proteobacteria.c_Alphaproteobacteria.o_Rhodobacterales.f_Rhodobacteraceae_unclassified	2.43	2.11	0.000037
Proteobacteria.c_Gammaproteobacteria.o_Legionellales.f_Legionellaceae.g_Tatlockia	1.65	2.11	0.000405
Armatimonadetes.c_Armatimonadia.o_Armatimonadales.f_Armatimonadaceae_unclassified	1.12	2.12	0.027012
Gemmatimonadetes.c_Gemmatimonadetes.o_Gemmatimonadales	2.56	2.17	0.000703
Gemmatimonadetes.c_Gemmatimonadetes	2.57	2.17	0.000799
Gemmatimonadetes.c_Gemmatimonadetes.o_Gemmatimonadales.f_Gemmatimonadaceae.g_Gemmatimonas	2.56	2.19	0.000566
Gemmatimonadetes.c_Gemmatimonadetes.o_Gemmatimonadales.f_Gemmatimonadaceae	2.56	2.19	0.000566
Proteobacteria.c_Alphaproteobacteria.o_Rhodospirillales.f_Rhodospirillaceae_unclassified	2.81	2.23	0.002217
Verrucomicrobia.c_Verrucomicrobiae.o_Verrucomicrobiales.f_Verrucomicrobiaceae.g_Prosthecobacter	2.60	2.31	0.000925

Verrucomicrobia.c___Spartobacteria_.o___Chthoniobacterales_.f___Chthoniobacteraceae_unclassified	2.63	2.34	0.000154
Verrucomicrobia.c_Verrucomicrobiae.o_Verrucomicrobiales.f_Verrucomicrobiaceae	2.67	2.35	0.001091
Verrucomicrobia.c_Verrucomicrobiae	2.84	2.51	0.000512
Verrucomicrobia.c_Verrucomicrobiae.o_Verrucomicrobiales	2.84	2.51	0.000512
Actinobacteria.c_Thermoleophilia	3.09	2.73	0.000127
Actinobacteria.c_Thermoleophilia.o_Gaiellales_unclassified	3.08	2.77	0.000005
Proteobacteria.c_Gammaproteobacteria.o_Alteromonadales.f_125ds10_unclassified	3.16	2.80	0.000001
Proteobacteria.c_Gammaproteobacteria.o_Methylococcales.f_Methylococcaceae.g_Methylocaldum	3.17	2.88	0.000003
Proteobacteria.c_Gammaproteobacteria.o_Methylococcales.f_Methylococcaceae	3.18	2.89	0.000003
Proteobacteria.c_Gammaproteobacteria.o_Methylococcales	3.19	2.90	0.000014
Proteobacteria.c_Alphaproteobacteria.o_Rhizobiales.f_Methylocystaceae.g_Methylosinus	3.25	2.91	0.000001
Proteobacteria.c_Alphaproteobacteria.o_Rhizobiales.f_Methylocystaceae	3.25	2.91	0.000001
Verrucomicrobia.c___Pedosphaerae_.o___Pedosphaerales_	3.28	2.95	0.000000
Verrucomicrobia.c___Pedosphaerae_	3.28	2.95	0.000000
Verrucomicrobia.c___Pedosphaerae_.o___Pedosphaerales_.f_R4_41B_unclassified	3.28	2.95	0.000000
Firmicutes.c_Bacilli.o_Bacillales.f_Bacillaceae.g_Bacillus	3.27	2.97	0.000623
Firmicutes.c_Bacilli.o_Bacillales.f_Bacillaceae	3.27	2.97	0.000623
Firmicutes.c_Bacilli.o_Bacillales	3.27	2.97	0.008123
Firmicutes.c_Bacilli	3.34	2.98	0.030747
Chlorobi	3.38	3.05	0.000037
Chlorobi.c_OPB56_unclassified	3.38	3.06	0.000003
Actinobacteria.c_Acidimicrobiia.o_Acidimicrobiales_unclassified	3.45	3.14	0.000009
Proteobacteria.c_Gammaproteobacteria.o_Pseudomonadales.f_Pseudomonadaceae	3.47	3.16	0.000008
Proteobacteria.c_Gammaproteobacteria.o_Pseudomonadales.f_Pseudomonadaceae.g_Pseudomonas	3.47	3.16	0.000008
Proteobacteria.c_TA18	3.48	3.18	0.003680
Proteobacteria.c_TA18.o_PHOS_HD29_unclassified	3.48	3.18	0.003496
Proteobacteria.c_Alphaproteobacteria.o_Sphingomonadales.f_Sphingomonadaceae_Other	3.49	3.20	0.000000
Proteobacteria.c_Gammaproteobacteria.o_Pseudomonadales	3.56	3.24	0.000274
Proteobacteria.c_Betaproteobacteria.o_Burkholderiales.f_Oxalobacteraceae_unclassified	3.64	3.24	0.000144

Proteobacteria.c_Betaproteobacteria.o_Rhodocyclales.f_Rhodocyclaceae_unclassified	3.85	3.28	0.028091
Proteobacteria.c_Gammaproteobacteria.o_Xanthomonadales.f_Sinobacteraceae_unclassified	3.70	3.30	0.000009
Proteobacteria.c_Gammaproteobacteria.o_Xanthomonadales	3.73	3.31	0.002211
Proteobacteria.c_Betaproteobacteria_unclassified	3.75	3.44	0.000004
Proteobacteria.c_Gammaproteobacteria.o_Chromatiales	3.73	3.46	0.000003
Proteobacteria.c_Gammaproteobacteria.o_Chromatiales.f_Halothiobacillaceae.g_Thiovirga	3.72	3.46	0.000002
Proteobacteria.c_Gammaproteobacteria.o_Chromatiales.f_Halothiobacillaceae	3.72	3.46	0.000002
Proteobacteria.c_Gammaproteobacteria.o_Alteromonadales.f_HTCC2188.g_HTCC	4.01	3.70	0.000001
Proteobacteria.c_Gammaproteobacteria.o_Alteromonadales.f_HTCC2188	4.01	3.70	0.000001
Proteobacteria.c_Alphaproteobacteria.o_Rhizobiales_unclassified	4.04	3.72	0.000029
Proteobacteria.c_Gammaproteobacteria.o_Alteromonadales	4.09	3.73	0.000053
Proteobacteria.c_Betaproteobacteria.o_Burkholderiales_unclassified	4.28	3.77	0.000001
Actinobacteria.c_Acidimicrobiia.o_Acidimicrobiales.f_C111_unclassified	4.42	4.07	0.000171
Actinobacteria.c_Acidimicrobiia.o_Acidimicrobiales	4.42	4.07	0.000173
Proteobacteria.c_Gammaproteobacteria	4.50	4.07	0.000126
Proteobacteria.c_Alphaproteobacteria.o_Rhodobacterales	4.54	4.08	0.000021
Proteobacteria.c_Alphaproteobacteria.o_Rhodobacterales.f_Rhodobacteraceae.g_Rhodobacter	4.54	4.08	0.000019
Proteobacteria.c_Alphaproteobacteria.o_Rhodobacterales.f_Rhodobacteraceae	4.54	4.08	0.000019
Actinobacteria.c_Acidimicrobiia	4.47	4.12	0.000066

JP

**Taxon**	**value**	**LDA score**	**P**
Proteobacteria.c_Gammaproteobacteria.o_Oceanospirillales	2.17	2.01	0.004954
Bacteroidetes.c_Bacteroidia.o_Bacteroidales.f_Rikenellaceae	2.26	2.01	0.000157
Firmicutes.c__Clostridia.o__Clostridiales.f___Acidaminobacteraceae_.g_Acidaminobacter	2.29	2.02	0.000002
WS5_unclassified	2.11	2.03	0.000024
Fibrobacteres.c_TG3	1.35	2.04	0.036520
OP3.c_PBS_25_unclassified	1.25	2.04	0.021740

Spirochaetes.c_Spirochaetes.o_Spirochaetales.f_Spirochaetaceae.g_Treponema	2.13	2.05	0.000017
Bacteroidetes.c_Bacteroidia.o_Bacteroidales.f_Rikenellaceae.g_Blvii28	2.26	2.06	0.000286
Actinobacteria.c_OPB41_unclassified	2.15	2.07	0.012655
Nitrospirae.c__Nitrospira.o__Nitrospirales.f___Thermodesulfovibrionaceae_.g_GOUTA19	2.32	2.08	0.000004
Proteobacteria.c_Gammaproteobacteria_Other_Other	1.93	2.08	0.003572
Firmicutes.c_Clostridia.o_Natranaerobiales.f_ML1228J_1_unclassified	2.15	2.08	0.000021
Proteobacteria.c_Alphaproteobacteria.o_Rhodobacterales.f_Hyphomonadaceae_Other	2.22	2.09	0.000046
Proteobacteria.c_Alphaproteobacteria.o_Rhodospirillales.f_Rhodospirillaceae.g_Telmatospirillum	2.20	2.10	0.001445
Proteobacteria.c_Gammaproteobacteria.o_Oceanospirillales.f_Oleiphilaceae_unclassified	1.55	2.10	0.012464
Proteobacteria.c_Deltaproteobacteria.o_Desulfovibrionales	2.18	2.10	0.000944
Spirochaetes.c_Spirochaetes.o_Spirochaetales.f_Spirochaetaceae	2.19	2.10	0.000037
WS3.c_PRR_12.o_GN03.f_KSB4_unclassified	1.13	2.10	0.032301
Acidobacteria.c_Acidobacteria_6.o_iii1_15	2.33	2.11	0.000092
UnclassifiedBacteria	1.55	2.11	0.004625
Proteobacteria.c_Gammaproteobacteria.o_Legionellales.f_Coxiellaceae_unclassified	2.41	2.12	0.000104
Chlorobi.c_Ignavibacteria.o_Ignavibacteriales.f_Ignavibacteriaceae_unclassified	2.28	2.12	0.000011
Chloroflexi.c_Anaerolineae	1.25	2.13	0.014077
Proteobacteria.c_Gammaproteobacteria.o_Alteromonadales.f_Alteromonadaceae_unclassified	2.32	2.13	0.001406
Proteobacteria.c_Alphaproteobacteria.o_Rhodobacterales.f_Hyphomonadaceae.g_Hyphomonas	2.45	2.15	0.000452
Proteobacteria.c_Alphaproteobacteria.o_Rhodobacterales.f__Hyphomonadaceae	2.45	2.15	0.000452
Spirochaetes.c_Spirochaetes.o_Spirochaetales	2.26	2.16	0.000005
Proteobacteria.c_Deltaproteobacteria.o_Desulfobacterales.f_Desulfobulbaceae_unclassified	2.40	2.16	0.001285
Bacteroidetes.c_Cytophagia.o_Cytophagales.f_Cytophagaceae.g_Leadbetterella	2.36	2.16	0.000004
Gemmatimonadetes.c_Gemmatimonadetes.o_KD8_87_unclassified	2.40	2.17	0.000092
Chlorobi.c_Ignavibacteria	2.34	2.17	0.000007
Chlorobi.c_Ignavibacteria.o_Ignavibacteriales	2.34	2.17	0.000007
Spirochaetes.c_Spirochaetes	2.27	2.17	0.000004
Proteobacteria.c_Betaproteobacteria.o_Rhodocyclales.f_Rhodocyclaceae.g_Sulfuritalea	2.40	2.17	0.000004
Bacteroidetes.c_Cytophagia.o_Cytophagales.f_Cytophagaceae	2.45	2.22	0.000267

Acidobacteria.c_Holophagae.o_Holophagales.f_Holophagaceae_unclassified	2.46	2.23	0.000012
Acidobacteria.c_Holophagae.o_Holophagales	2.47	2.24	0.000013
Acidobacteria.c_Holophagae	2.47	2.24	0.000013
WS3.c_PRR_12.o_GN03	1.13	2.25	0.032301
Nitrospirae.c__Nitrospira.o__Nitrospirales.f___Thermodesulfovibrionaceae_	2.53	2.28	0.000011
Proteobacteria.c_Deltaproteobacteria.o_Desulfuromonadales.f_Geobacteraceae.g_Geobacter	2.52	2.34	0.000004
Proteobacteria.c_Deltaproteobacteria.o_Desulfuromonadales.f_Geobacteraceae	2.52	2.34	0.000004
Proteobacteria.c__Deltaproteobacteria.o___Entotheonellales_unclassified	2.60	2.36	0.000003
Proteobacteria.c_Alphaproteobacteria.o_Caulobacterales.f_Caulobacteraceae	2.77	2.37	0.009258
Spirochaetes	2.71	2.37	0.034600
Gemmatimonadetes	2.69	2.41	0.000258
Proteobacteria.c_Alphaproteobacteria.o_Caulobacterales.f_Caulobacteraceae.g_Mycoplana	2.70	2.41	0.000009
Firmicutes.c__Clostridia.o__Clostridiales.f___Acidaminobacteraceae_.g_Fusibacter	2.58	2.43	0.000036
Acidobacteria.c_Acidobacteria_6	2.70	2.43	0.000014
Proteobacteria.c_Deltaproteobacteria.o_Desulfobacterales	2.69	2.43	0.000069
Proteobacteria.c_Deltaproteobacteria_unclassified	2.68	2.43	0.000000
Proteobacteria.c_Betaproteobacteria.o_Ellin6067_unclassified	2.73	2.44	0.000000
Proteobacteria.c_Alphaproteobacteria.o_Rhodobacterales.f_Hyphomonadaceae_unclassified	2.84	2.45	0.000001
Bacteroidetes.c_Bacteroidia.o_Bacteroidales_unclassified	2.61	2.46	0.000001
Proteobacteria.c_Deltaproteobacteria.o_Desulfuromonadales	2.71	2.55	0.000003
Firmicutes.c_Clostridia.o_Natranaerobiales	2.59	2.55	0.000021
Firmicutes.c__Clostridia.o__Clostridiales.f___Acidaminobacteraceae_	2.76	2.56	0.000000
Firmicutes.c_Clostridia.o_Thermoanaerobacterales_unclassified	2.74	2.68	0.042285
TM7.c_TM7_1_unclassified	2.95	2.72	0.000000
TM7	2.99	2.76	0.000000
Proteobacteria.c_Betaproteobacteria.o_Rhodocyclales.f_Rhodocyclaceae_Other	3.00	2.76	0.000063
Acidobacteria	3.07	2.79	0.000020
Proteobacteria.c_Gammaproteobacteria.o_Aeromonadales	3.22	2.79	0.000593
Proteobacteria.c_Gammaproteobacteria.o_Aeromonadales.f_Aeromonadaceae_unclassified	3.21	2.80	0.000427

Proteobacteria.c_Deltaproteobacteria.o_Bdellovibrionales.f_Bdellovibrionaceae.g_Bdellovibrio	3.13	2.86	0.000000
Proteobacteria.c_Deltaproteobacteria.o_Bdellovibrionales.f_Bdellovibrionaceae	3.13	2.86	0.000000
Proteobacteria.c_Alphaproteobacteria_unclassified	3.24	2.93	0.000000
Bacteroidetes.c_Bacteroidia.o_Bacteroidales.f_ML635J_40_unclassified	3.32	3.29	0.000976
Bacteroidetes.c_Bacteroidia.o_Bacteroidales	3.53	3.36	0.007644
Proteobacteria.c_Deltaproteobacteria	3.82	3.39	0.000568
Bacteroidetes.c_Bacteroidia	3.58	3.41	0.000862
Proteobacteria.c_Alphaproteobacteria.o_Caulobacterales.f_Caulobacteraceae_unclassified	3.76	3.44	0.000014
Proteobacteria.c_Alphaproteobacteria.o_Caulobacterales	3.80	3.48	0.000009
Bacteroidetes.c_Flavobacteriia.o_Flavobacteriales.f_Cryomorphaceae_unclassified	4.33	3.70	0.006891
Bacteroidetes.c_Sphingobacteriia	4.60	4.22	0.000042
Bacteroidetes.c_Sphingobacteriia.o_Sphingobacteriales_unclassified	4.58	4.22	0.000066

Supplemental Table 4: LEfSe results of a comparison of only the nearshore Lake Michigan sampling sites, taxa at all levels with LDA >2.0 are listed

GCM

**Taxon**	**value**	**LDA**	**P**
Acidobacteria.c_Solibacteres.o_Solibacterales	2.19	2.06	0.00287
Acidobacteria.c__Solibacteres.o__Solibacterales.f___Bryobacteraceae_unclassified	2.15	2.09	0.00133
Actinobacteria.c_Acidimicrobiia	4.47	3.78	0.02534
Actinobacteria.c_Acidimicrobiia.o_Acidimicrobiales_unclassified	3.45	3.09	0.00005
Actinobacteria.c_Actinobacteria.o_Actinomycetales.f_Microbacteriaceae	3.69	3.29	0.00006
Actinobacteria.c_Actinobacteria.o_Actinomycetales.f_Microbacteriaceae.g_CandidatusAquiluna	3.69	3.29	0.00009
Actinobacteria.c_Actinobacteria.o_Actinomycetales.f_Microbacteriaceae.g_Clavibacter	1.35	2.48	0.01648
Actinobacteria.c_Thermoleophilia	3.09	2.73	0.00021
Actinobacteria.c_Thermoleophilia.o_Gaiellales_unclassified	3.08	2.78	0.00012
Armatimonadetes.c_Armatimonadia	1.12	2.64	0.02308
Armatimonadetes.c_Armatimonadia.o_Armatimonadales	1.12	2.70	0.02308
Armatimonadetes.c_Armatimonadia.o_Armatimonadales.f_Armatimonadaceae_unclassified	1.12	2.63	0.02308

Bacteroidetes.c_Cytophagia.o_Cytophagales.f_Cytophagaceae.g_Flectobacillus	1.65	2.20	0.00012
Bacteroidetes.c_Flavobacteriia.o_Flavobacteriales.f_Cryomorphaceae	4.46	3.92	0.03170
Bacteroidetes.c_Flavobacteriia.o_Flavobacteriales.f_Cryomorphaceae.g_Fluviicola	4.46	3.92	0.03170
Chlorobi	3.38	3.04	0.00011
Chlorobi.c_OPB56_unclassified	3.38	3.07	0.00006
Chloroflexi.c_Chloroflexi.o_Chloroflexales	1.25	2.46	0.02308
Chloroflexi.c_Chloroflexi.o_Chloroflexales.f_Chloroflexaceae_Other	1.25	2.29	0.02308
Firmicutes.c_Bacilli.o_Bacillales_unclassified	1.85	2.13	0.02308
Gemmatimonadetes.c_Gemmatimonadetes.o_Gemmatimonadales	2.56	2.20	0.00111
Gemmatimonadetes.c_Gemmatimonadetes.o_Gemmatimonadales.f_Gemmatimonadaceae	2.56	2.22	0.00057
Gemmatimonadetes.c_Gemmatimonadetes.o_Gemmatimonadales.f_Gemmatimonadaceae.g_Gemmatimonas	2.56	2.22	0.00057
Proteobacteria	5.69	4.64	0.00017
Proteobacteria.c_Alphaproteobacteria.o_Caulobacterales.f_Caulobacteraceae.g_Phenylobacterium	2.29	2.17	0.02088
Proteobacteria.c_Alphaproteobacteria.o_Ellin329_unclassified	1.65	2.23	0.02110
Proteobacteria.c_Alphaproteobacteria.o_Rhizobiales	3.43	2.94	0.00000
Proteobacteria.c_Alphaproteobacteria.o_Rhizobiales_unclassified	4.04	3.49	0.00308
Proteobacteria.c_Alphaproteobacteria.o_Rhizobiales.f_Methylocystaceae	3.25	2.90	0.00016
Proteobacteria.c_Alphaproteobacteria.o_Rhizobiales.f_Methylocystaceae.g_Methylosinus	3.25	2.90	0.00016
Proteobacteria.c_Alphaproteobacteria.o_Rhizobiales.f_Rhizobiaceae_unclassified	1.97	2.31	0.01690
Proteobacteria.c_Alphaproteobacteria.o_Rhizobiales.f_Xanthobacteraceae	1.95	2.10	0.00001
Proteobacteria.c_Alphaproteobacteria.o_Rhizobiales.f_Xanthobacteraceae_Other	1.35	2.47	0.00448
Proteobacteria.c_Alphaproteobacteria.o_Rhizobiales.f_Xanthobacteraceae.g_Xanthobacter	1.95	2.09	0.00001
Proteobacteria.c_Alphaproteobacteria.o_Rhodobacterales	4.54	4.07	0.00005
Proteobacteria.c_Alphaproteobacteria.o_Rhodobacterales.f_Rhodobacteraceae	4.54	4.08	0.00004
Proteobacteria.c_Alphaproteobacteria.o_Rhodobacterales.f_Rhodobacteraceae_unclassified	2.43	2.28	0.00001
Proteobacteria.c_Alphaproteobacteria.o_Rhodobacterales.f_Rhodobacteraceae.g_Rhodobacter	4.54	4.08	0.00004
Proteobacteria.c_Alphaproteobacteria.o_Rhodospirillales.f_Acetobacteraceae	1.60	2.35	0.02185
Proteobacteria.c_Alphaproteobacteria.o_Rhodospirillales.f_Acetobacteraceae.g_Roseococcus	1.60	2.34	0.02185
Proteobacteria.c_Alphaproteobacteria.o_Sphingomonadales.f_Erythrobacteraceae_unclassified	3.06	2.74	0.00004

Proteobacteria.c_Alphaproteobacteria.o_Sphingomonadales.f_Sphingomonadaceae	3.83	3.49	0.00001
Proteobacteria.c_Alphaproteobacteria.o_Sphingomonadales.f_Sphingomonadaceae_Other	3.49	3.20	0.00001
Proteobacteria.c_Alphaproteobacteria.o_Sphingomonadales.f_Sphingomonadaceae.g_Novosphingobium	3.81	3.49	0.00002
Proteobacteria.c_Betaproteobacteria	5.47	4.39	0.00543
Proteobacteria.c_Betaproteobacteria_unclassified	3.75	3.42	0.00005
Proteobacteria.c_Betaproteobacteria.o_Burkholderiales_unclassified	4.28	3.77	0.00030
Proteobacteria.c_Betaproteobacteria.o_Burkholderiales.f_Comamonadaceae_Other	4.74	4.40	0.00000
Proteobacteria.c_Betaproteobacteria.o_Burkholderiales.f_Comamonadaceae_unclassified	4.72	3.96	0.00577
Proteobacteria.c_Betaproteobacteria.o_Burkholderiales.f_Comamonadaceae.g_Hydrogenophaga	3.59	2.99	0.00690
Proteobacteria.c_Betaproteobacteria.o_Burkholderiales.f_Comamonadaceae.g_Roseateles	2.40	2.27	0.00055
Proteobacteria.c_Betaproteobacteria.o_Burkholderiales.f_Comamonadaceae.g_Tepidimonas	1.43	2.50	0.00448
Proteobacteria.c_Betaproteobacteria.o_Burkholderiales.f_Comamonadaceae.g_Variovorax	1.49	2.48	0.04131
Proteobacteria.c_Betaproteobacteria.o_Burkholderiales.f_Oxalobacteraceae	3.90	3.58	0.00001
Proteobacteria.c_Betaproteobacteria.o_Burkholderiales.f_Oxalobacteraceae.g_Polynucleobacter	3.90	3.57	0.00001
Proteobacteria.c_Betaproteobacteria.o_Methylophilales.f_Methylophilaceae	1.90	2.27	0.03266
Proteobacteria.c_Betaproteobacteria.o_Methylophilales.f_Methylophilaceae.g_Methylotenera	1.90	2.26	0.03266
Proteobacteria.c_Betaproteobacteria.o_MWH_UniP1_unclassified	3.21	2.56	0.02465
Proteobacteria.c_Betaproteobacteria.o_Rhodocyclales.f_Rhodocyclaceae.g_C39	2.28	2.17	0.00231
Proteobacteria.c_Betaproteobacteria.o_Rhodocyclales.f_Rhodocyclaceae.g_Hydrogenophilus	1.90	2.11	0.02308
Proteobacteria.c_Deltaproteobacteria.o_Bdellovibrionales.f_Bacteriovoracaceae_unclassified	3.00	2.45	0.02374
Proteobacteria.c_Deltaproteobacteria.o_Myxococcales.f_Myxococcaceae_unclassified	2.03	2.01	0.00412
Proteobacteria.c_Gammaproteobacteria	4.50	4.08	0.00025
Proteobacteria.c_Gammaproteobacteria.o_34P16_unclassified	2.24	2.26	0.00020
Proteobacteria.c_Gammaproteobacteria.o_Aeromonadales.f_Aeromonadaceae.g_Aeromonas	1.35	2.80	0.00449
Proteobacteria.c_Gammaproteobacteria.o_Alteromonadales	4.09	3.74	0.00016
Proteobacteria.c_Gammaproteobacteria.o_Alteromonadales.f_125ds10_unclassified	3.16	2.80	0.00003
Proteobacteria.c_Gammaproteobacteria.o_Alteromonadales.f_Alteromonadaceae.g_HB2_32_21	1.93	2.36	0.00099
Proteobacteria.c_Gammaproteobacteria.o_Alteromonadales.f_HTCC2188	4.01	3.70	0.00005
Proteobacteria.c_Gammaproteobacteria.o_Alteromonadales.f_HTCC2188_unclassified	2.06	2.06	0.00693

Proteobacteria.c_Gammaproteobacteria.o_Alteromonadales.f_HTCC2188.g_HTCC	4.01	3.70	0.00005
Proteobacteria.c_Gammaproteobacteria.o_Chromatiales	3.73	3.45	0.00007
Proteobacteria.c_Gammaproteobacteria.o_Chromatiales_unclassified	3.13	2.63	0.02244
Proteobacteria.c_Gammaproteobacteria.o_Chromatiales.f_Ectothiorhodospiraceae_unclassified	1.25	2.31	0.02827
Proteobacteria.c_Gammaproteobacteria.o_Chromatiales.f_Halothiobacillaceae	3.72	3.45	0.00009
Proteobacteria.c_Gammaproteobacteria.o_Chromatiales.f_Halothiobacillaceae.g_Thiovirga	3.72	3.45	0.00009
Proteobacteria.c_Gammaproteobacteria.o_Legionellales.f_Legionellaceae_unclassified	2.63	2.19	0.03882
Proteobacteria.c_Gammaproteobacteria.o_Legionellales.f_Legionellaceae.g_Tatlockia	1.65	2.41	0.00575
Proteobacteria.c_Gammaproteobacteria.o_Methylococcales	3.19	2.89	0.00005
Proteobacteria.c_Gammaproteobacteria.o_Methylococcales.f_Methylococcaceae	3.18	2.88	0.00002
Proteobacteria.c_Gammaproteobacteria.o_Methylococcales.f_Methylococcaceae.g_Methylocaldum	3.17	2.88	0.00000
Proteobacteria.c_Gammaproteobacteria.o_Methylococcales.f_Methylococcaceae.g_Methylomonas	1.60	2.43	0.00563
Proteobacteria.c_Gammaproteobacteria.o_Pseudomonadales	3.56	3.23	0.00099
Proteobacteria.c_Gammaproteobacteria.o_Pseudomonadales.f_Moraxellaceae	2.72	2.45	0.00990
Proteobacteria.c_Gammaproteobacteria.o_Pseudomonadales.f_Moraxellaceae.g_Perlucidibaca	2.33	2.25	0.00001
Proteobacteria.c_Gammaproteobacteria.o_Pseudomonadales.f_Pseudomonadaceae	3.47	3.13	0.00024
Proteobacteria.c_Gammaproteobacteria.o_Pseudomonadales.f_Pseudomonadaceae.g_Pseudomonas	3.47	3.13	0.00024
Proteobacteria.c_Gammaproteobacteria.o_PYR10d3_unclassified	2.01	2.37	0.00009
Proteobacteria.c_Gammaproteobacteria.o_Xanthomonadales.f_Sinobacteraceae_unclassified	3.70	3.25	0.00090
Proteobacteria.c_TA18	3.48	3.03	0.02039
Proteobacteria.c_TA18.o_PHOS_HD29_unclassified	3.48	3.06	0.01655
Verrucomicrobia.c___Pedosphaerae_	3.28	2.86	0.00021
Verrucomicrobia.c___Pedosphaerae_.o___Pedosphaerales_	3.28	2.87	0.00020
Verrucomicrobia.c___Pedosphaerae_.o___Pedosphaerales_.f_R4_41B_unclassified	3.28	2.87	0.00021
Verrucomicrobia.c___Spartobacteria_	2.95	2.45	0.03571
Verrucomicrobia.c___Spartobacteria_.o___Chthoniobacterales_	2.95	2.45	0.03571
Verrucomicrobia.c___Spartobacteria_.o___Chthoniobacterales_.f___Chthoniobacteraceae_unclassified	2.63	2.34	0.00008
Verrucomicrobia.c__Opitutae.o___Cerasicoccales_	1.85	2.06	0.00045
Verrucomicrobia.c__Opitutae.o___Cerasicoccales_.f___Cerasicoccaceae_unclassified	1.85	2.07	0.00045

Verrucomicrobia.c_Verrucomicrobiae	2.84	2.54	0.00017
Verrucomicrobia.c_Verrucomicrobiae.o_Verrucomicrobiales	2.84	2.54	0.00017
Verrucomicrobia.c_Verrucomicrobiae.o_Verrucomicrobiales.f_Verrucomicrobiaceae	2.67	2.38	0.00025
Verrucomicrobia.c_Verrucomicrobiae.o_Verrucomicrobiales.f_Verrucomicrobiaceae_unclassified	2.34	2.26	0.00022
Verrucomicrobia.c_Verrucomicrobiae.o_Verrucomicrobiales.f_Verrucomicrobiaceae.g_Prosthecobacter	2.60	2.38	0.00017

JP

**Taxon**	**value**	**LDA**	**P**
Acidobacteria	3.07	2.62	0.0300258
Acidobacteria.c_Acidobacteria_6	2.70	2.34	0.0088230
Acidobacteria.c_Acidobacteria_6.o_iii1_15	2.33	2.08	0.0144007
Acidobacteria.c_Holophagae	2.47	2.21	0.0014101
Acidobacteria.c_Holophagae.o_Holophagales	2.47	2.21	0.0014101
Acidobacteria.c_Holophagae.o_Holophagales.f_Holophagaceae_unclassified	2.46	2.20	0.0013513
Actinobacteria.c_Thermoleophilia.o_Gaiellales	2.11	2.02	0.0027531
Bacteroidetes.c_Bacteroidia.o_Bacteroidales_unclassified	2.61	2.33	0.0035442
Bacteroidetes.c_Bacteroidia.o_Bacteroidales.f_ML635J_40_unclassified	3.32	3.07	0.0221840
Bacteroidetes.c_Bacteroidia.o_Bacteroidales.f_Porphyromonadaceae	2.39	2.12	0.0016399
Bacteroidetes.c_Bacteroidia.o_Bacteroidales.f_Porphyromonadaceae.g_Paludibacter	2.36	2.09	0.0073587
Bacteroidetes.c_Bacteroidia.o_Bacteroidales.f_Rikenellaceae	2.26	2.07	0.0042599
Bacteroidetes.c_Bacteroidia.o_Bacteroidales.f_Rikenellaceae.g_Blvii28	2.26	2.01	0.0050417
Bacteroidetes.c_Bacteroidia.o_Bacteroidales.f_SB_1_unclassified	1.76	2.16	0.0224758
Bacteroidetes.c_Cytophagia.o_Cytophagales.f_Cytophagaceae	2.45	2.14	0.0020970
Bacteroidetes.c_Cytophagia.o_Cytophagales.f_Cytophagaceae.g_Leadbetterella	2.36	2.09	0.0000321
Bacteroidetes.c_Flavobacteriia.o_Flavobacteriales.f_Cryomorphaceae_Other	1.65	2.23	0.0377649
Bacteroidetes.c_Flavobacteriia.o_Flavobacteriales.f_Cryomorphaceae_unclassified	4.33	3.66	0.0181039
Bacteroidetes.c_Sphingobacteriia	4.60	4.00	0.0024593
Bacteroidetes.c_Sphingobacteriia.o_Sphingobacteriales_unclassified	4.58	4.02	0.0021439
Chlorobi.c_Ignavibacteria	2.34	2.08	0.0011306

Chlorobi.c_Ignavibacteria.o_Ignavibacteriales	2.34	2.08	0.0011306
Chlorobi.c_Ignavibacteria.o_Ignavibacteriales.f_Ignavibacteriaceae_unclassified	2.28	2.03	0.0008642
Elusimicrobia.c_Endomicrobia_unclassified	1.65	2.02	0.0482427
Firmicutes.c__Clostridia.o__Clostridiales.f___Acidaminobacteraceae_	2.76	2.46	0.0000297
Firmicutes.c__Clostridia.o__Clostridiales.f___Acidaminobacteraceae_.g_Acidaminobacter	2.29	2.09	0.0001500
Firmicutes.c__Clostridia.o__Clostridiales.f___Acidaminobacteraceae_.g_Fusibacter	2.58	2.32	0.0002624
Firmicutes.c_Clostridia.o_Natranaerobiales	2.59	2.40	0.0007693
Firmicutes.c_Clostridia.o_Natranaerobiales.f_ML1228J_1_unclassified	2.15	2.07	0.0007693
Gemmatimonadetes.c_Gemmatimonadetes.o_KD8_87_unclassified	2.40	2.13	0.0017213
GN02_unclassified	1.25	2.50	0.0342774
Nitrospirae	2.84	2.51	0.0009425
Nitrospirae.c_Nitrospira	2.84	2.51	0.0009425
Nitrospirae.c_Nitrospira.o_Nitrospirales	2.84	2.51	0.0009425
Nitrospirae.c__Nitrospira.o__Nitrospirales.f___Thermodesulfovibrionaceae_	2.53	2.24	0.0051347
Nitrospirae.c__Nitrospira.o__Nitrospirales.f___Thermodesulfovibrionaceae_.g_GOUTA19	2.32	2.05	0.0011777
Planctomycetes.c_vadinHA49	1.73	2.04	0.0055760
Planctomycetes.c_vadinHA49.o_DH61_unclassified	1.43	2.26	0.0218820
Proteobacteria_unclassified	2.11	2.06	0.0034372
Proteobacteria.c_Alphaproteobacteria_unclassified	3.24	2.81	0.0003380
Proteobacteria.c_Alphaproteobacteria.o_Caulobacterales	3.80	3.13	0.0020532
Proteobacteria.c_Alphaproteobacteria.o_Caulobacterales.f_Caulobacteraceae_unclassified	3.76	3.08	0.0033485
Proteobacteria.c_Alphaproteobacteria.o_Caulobacterales.f_Caulobacteraceae.g_Mycoplana	2.70	2.28	0.0084075
Proteobacteria.c_Alphaproteobacteria.o_Rhizobiales.f_Beijerinckiaceae_Other	2.94	2.47	0.0011745
Proteobacteria.c_Alphaproteobacteria.o_Rhodobacterales.f_Hyphomonadaceae	2.45	2.06	0.0112959
Proteobacteria.c_Alphaproteobacteria.o_Rhodobacterales.f_Hyphomonadaceae_unclassified	2.84	2.36	0.0003477
Proteobacteria.c_Alphaproteobacteria.o_Rhodobacterales.f_Hyphomonadaceae.g_Hyphomonas	2.45	2.06	0.0112959
Proteobacteria.c_Alphaproteobacteria.o_Rhodospirillales.f_Acetobacteraceae_Other	2.60	2.05	0.0067568
Proteobacteria.c_Alphaproteobacteria.o_Sphingomonadales.f_Sphingomonadaceae.g_Sphingopyxis	1.55	2.19	0.0416077
Proteobacteria.c_Betaproteobacteria.o_Ellin6067_unclassified	2.73	2.40	0.0004323

Proteobacteria.c_Betaproteobacteria.o_Gallionellales	1.35	2.59	0.0429901
Proteobacteria.c_Betaproteobacteria.o_Gallionellales.f_Gallionellaceae	1.35	2.60	0.0429901
Proteobacteria.c_Betaproteobacteria.o_Gallionellales.f_Gallionellaceae.g_Gallionella	1.35	2.57	0.0429901
Proteobacteria.c_Betaproteobacteria.o_Neisseriales.f_Neisseriaceae_unclassified	2.35	2.09	0.0025798
Proteobacteria.c_Betaproteobacteria.o_Rhodocyclales.f_Rhodocyclaceae_Other	3.00	2.62	0.0449308
Proteobacteria.c_Betaproteobacteria.o_Rhodocyclales.f_Rhodocyclaceae.g_Azospira	1.82	2.09	0.0000177
Proteobacteria.c_Betaproteobacteria.o_Rhodocyclales.f_Rhodocyclaceae.g_Dechloromonas	2.78	2.41	0.0052049
Proteobacteria.c_Deltaproteobacteria_unclassified	2.68	2.35	0.0004416
Proteobacteria.c__Deltaproteobacteria.o___Entotheonellales_unclassified	2.60	2.34	0.0010092
Proteobacteria.c_Deltaproteobacteria.o_Bdellovibrionales.f_Bdellovibrionaceae	3.13	2.68	0.0007973
Proteobacteria.c_Deltaproteobacteria.o_Bdellovibrionales.f_Bdellovibrionaceae.g_Bdellovibrio	3.13	2.68	0.0007973
Proteobacteria.c_Deltaproteobacteria.o_Desulfobacterales	2.69	2.39	0.0019610
Proteobacteria.c_Deltaproteobacteria.o_Desulfobacterales.f_Desulfobulbaceae_unclassified	2.40	2.12	0.0086299
Proteobacteria.c_Deltaproteobacteria.o_Desulfobacterales.f_Desulfobulbaceae.g_Desulfobulbus	1.60	2.00	0.0303988
Proteobacteria.c_Deltaproteobacteria.o_Desulfuromonadales	2.71	2.43	0.0006081
Proteobacteria.c_Deltaproteobacteria.o_Desulfuromonadales.f_Geobacteraceae	2.52	2.25	0.0008289
Proteobacteria.c_Deltaproteobacteria.o_Desulfuromonadales.f_Geobacteraceae.g_Geobacter	2.52	2.25	0.0008289
Proteobacteria.c_Deltaproteobacteria.o_Desulfuromonadales.f_Pelobacteraceae_unclassified	2.05	2.11	0.0007693
Proteobacteria.c_Epsilonproteobacteria.o_Campylobacterales.f_Campylobacteraceae.g_Sulfurospirillum	1.35	2.19	0.0266885
Proteobacteria.c_Gammaproteobacteria_Other_Other	1.93	2.13	0.0076455
Proteobacteria.c_Gammaproteobacteria.o_Aeromonadales	3.22	2.63	0.0252480
Proteobacteria.c_Gammaproteobacteria.o_Aeromonadales.f_Aeromonadaceae_unclassified	3.21	2.65	0.0178804
Proteobacteria.c_Gammaproteobacteria.o_Alteromonadales.f_Alteromonadaceae_unclassified	2.32	2.02	0.0317263
Proteobacteria.c_Gammaproteobacteria.o_Enterobacteriales	2.33	2.00	0.0181694
Proteobacteria.c_Gammaproteobacteria.o_Enterobacteriales.f_Enterobacteriaceae_unclassified	2.17	2.01	0.0002306
Proteobacteria.c_Gammaproteobacteria.o_Oceanospirillales.f_Oleiphilaceae_unclassified	1.55	2.06	0.0412118
Spirochaetes	2.71	2.32	0.0475323
Spirochaetes.c_Spirochaetes	2.27	2.09	0.0092093
Spirochaetes.c_Spirochaetes.o_Spirochaetales	2.26	2.10	0.0113581

Spirochaetes.c_Spirochaetes.o_Spirochaetales.f_Spirochaetaceae	2.19	2.08	0.0455015
Spirochaetes.c_Spirochaetes.o_Spirochaetales.f_Spirochaetaceae.g_Treponema	2.13	2.11	0.0187665
SR1_unclassified	2.35	2.07	0.0009415
TM7	2.99	2.65	0.0007457
TM7.c_TM7_1_unclassified	2.95	2.61	0.0009668
Unclassified Bacteria	1.55	2.22	0.0173974

Site 63

**Taxon**	**value**	**LDA**	**P**
Acidobacteria.c_Acidobacteria_6.o_iii1_15.f_mb2424_unclassified	2.25	2.06	0.021366
Acidobacteria.c_RB25_unclassified	2.30	2.05	0.001329
Actinobacteria	5.53	4.62	0.000153
Actinobacteria.c_Actinobacteria	5.51	4.68	0.000039
Actinobacteria.c_Actinobacteria.o_Actinomycetales	5.48	4.66	0.000041
Actinobacteria.c_Actinobacteria.o_Actinomycetales_unclassified	4.24	3.45	0.046281
Actinobacteria.c_Actinobacteria.o_Actinomycetales.f_Microbacteriaceae_Other	4.14	3.51	0.007084
Actinobacteria.c_Actinobacteria.o_Actinomycetales.f_Microbacteriaceae_unclassified	4.60	4.06	0.000084
Bacteroidetes.c_Sphingobacteriia.o_Sphingobacteriales	3.34	2.88	0.000213
Bacteroidetes.c_Sphingobacteriia.o_Sphingobacteriales.f_Sphingobacteriaceae_unclassified	3.34	2.88	0.000199
Chlorobi.c_BSV26.o_VC38_unclassified	1.49	2.06	0.038213
Chloroflexi.c_Ellin6529_unclassified	2.55	2.26	0.000425
Elusimicrobia	2.59	2.30	0.012827
Elusimicrobia.c_Elusimicrobia	2.55	2.26	0.018289
Elusimicrobia.c_Elusimicrobia.o_Elusimicrobiales_unclassified	2.26	2.07	0.021538
Fusobacteria	2.30	2.03	0.000126
Fusobacteria.c_Fusobacteriia	2.30	2.03	0.000126
Fusobacteria.c_Fusobacteriia.o_Fusobacteriales	1.82	2.13	0.039864
Fusobacteria.c_Fusobacteriia.o_Fusobacteriales.f_Fusobacteriaceae	1.82	2.13	0.039864
Nitrospirae.c_Nitrospira.o_Nitrospirales.f_FW	1.88	2.02	0.008172

Nitrospirae.c_Nitrospira.o_Nitrospirales.f_FW.g_4_29	1.88	2.03	0.008172
Proteobacteria.c_Alphaproteobacteria.o_BD7_3_unclassified	2.93	2.55	0.000345
Proteobacteria.c_Alphaproteobacteria.o_Rhodospirillales.f_Rhodospirillaceae.g_Azospirillum	1.69	2.21	0.002255
Proteobacteria.c_Alphaproteobacteria.o_Rickettsiales.f_Pelagibacteraceae_unclassified	3.53	2.96	0.015058
Proteobacteria.c_Betaproteobacteria.o_Burkholderiales	5.40	4.25	0.019303
Proteobacteria.c_Betaproteobacteria.o_Burkholderiales.f_Comamonadaceae	5.17	4.36	0.003937
Proteobacteria.c_Betaproteobacteria.o_Burkholderiales.f_Comamonadaceae.g_Leptothrix	1.60	2.18	0.049661
Proteobacteria.c_Betaproteobacteria.o_Burkholderiales.f_Comamonadaceae.g_Limnohabitans	5.09	4.31	0.007616
Proteobacteria.c_Betaproteobacteria.o_Burkholderiales.f_Comamonadaceae.g_Rhodoferax	4.26	3.61	0.030624
Proteobacteria.c_Betaproteobacteria.o_Burkholderiales.f_Oxalobacteraceae_Other	4.67	4.08	0.000378
Proteobacteria.c_Betaproteobacteria.o_Methylophilales_unclassified	1.43	2.47	0.023077
Proteobacteria.c_Betaproteobacteria.o_Neisseriales	2.84	2.51	0.000173
Proteobacteria.c_Betaproteobacteria.o_Neisseriales.f_Neisseriaceae	2.60	2.33	0.000755
Proteobacteria.c_Betaproteobacteria.o_Neisseriales.f_Neisseriaceae.g_Deefgea	2.53	2.31	0.001261
Proteobacteria.c_Betaproteobacteria.o_Rhodocyclales.f_Rhodocyclaceae.g_Propionivibrio	1.76	2.30	0.008487
Proteobacteria.c_Betaproteobacteria.o_SC_I_84_unclassified	1.82	2.00	0.022945
Proteobacteria.c_Deltaproteobacteria.o_Bdellovibrionales.f_Bdellovibrionaceae_unclassified	1.82	2.32	0.004591
Proteobacteria.c_Deltaproteobacteria.o_Myxococcales_unclassified	2.55	2.15	0.047019
Proteobacteria.c_Deltaproteobacteria.o_Myxococcales.f_Haliangiaceae_unclassified	1.60	2.03	0.022178
Proteobacteria.c_Deltaproteobacteria.o_NB1_j	1.76	2.04	0.002371
Proteobacteria.c_Deltaproteobacteria.o_NB1_j.f_JTB38_unclassified	1.69	2.06	0.015930
Proteobacteria.c_Deltaproteobacteria.o_Spirobacillales_unclassified	3.14	2.75	0.000211
Proteobacteria.c_Gammaproteobacteria.o_HTCC2188_unclassified	1.93	2.06	0.001908
Proteobacteria.c_Gammaproteobacteria.o_Legionellales.f_Coxiellaceae	2.34	2.04	0.002620
Proteobacteria.c_Gammaproteobacteria.o_Legionellales.f_Coxiellaceae.g_Aquicella	2.32	2.09	0.001127
Proteobacteria.c_Gammaproteobacteria.o_Methylococcales_Other	1.43	2.52	0.004491
Proteobacteria.c_Gammaproteobacteria.o_Thiotrichales	2.01	2.01	0.028996
Proteobacteria.c_Gammaproteobacteria.o_Thiotrichales.f_Piscirickettsiaceae_unclassified	2.01	2.02	0.018608
Proteobacteria.c_Gammaproteobacteria.o_Xanthomonadales.f_Xanthomonadaceae_unclassified	3.27	2.85	0.002087

Proteobacteria.c_TA18.o_CV90_unclassified	2.18	2.05	0.000544
TM6	2.41	2.10	0.005695
TM6.c_SBRH58_unclassified	2.05	2.13	0.012677
WS3	2.27	2.05	0.000262
WS3.c_PRR_12	2.27	2.05	0.000257

WHW

**Taxon**	**value**	**LDA**	**P**
Actinobacteria.c_Actinobacteria.o_Actinomycetales.f_ACK_M1_unclassified	5.40	4.53	0.000320
Actinobacteria.c_Actinobacteria.o_Actinomycetales.f_Cellulomonadaceae	1.55	2.36	0.035140
Actinobacteria.c_Actinobacteria.o_Actinomycetales.f_Nocardioidaceae.g_Nocardioides	1.49	2.70	0.019039
GN02.c_3BR_5F_unclassified	2.05	2.01	0.006166
Nitrospirae.c_Nitrospira.o_Nitrospirales.f_Nitrospiraceae	2.37	2.14	0.000596
Nitrospirae.c_Nitrospira.o_Nitrospirales.f_Nitrospiraceae.g_Nitrospira	2.37	2.14	0.000596
Proteobacteria.c_Alphaproteobacteria.o_Rickettsiales	3.85	3.28	0.002973
Proteobacteria.c_Alphaproteobacteria.o_Rickettsiales_unclassified	4.10	3.68	0.024571
Proteobacteria.c_Alphaproteobacteria.o_Sphingomonadales_unclassified	4.63	4.03	0.010236
Proteobacteria.c_Gammaproteobacteria.o_Legionellales_unclassified	2.49	2.33	0.000115
Proteobacteria.c_Gammaproteobacteria.o_Legionellales.f_Francisellaceae_Other	1.43	2.63	0.029783

Table S5: Environmental conditions and *E. coli* most probable numbers (MPN) in triplicate samples from each sampling location on three collection dates

#SampleID	Location	Date	Event	E. coli MPN	E. coli bin	Turbidity	Turbidity Bin	Water Temp (°C)	DO (mg/L)
W01	63	8/12/15	Dry	11	2	4.7	3	23.3	8.46
W02	63	8/12/15	Dry	9	2	4.8	3	23.3	8.46
W03	63	8/12/15	Dry	5	2	4.5	3	23.3	8.46
W04	GCR	8/12/15	Dry	17	2	2.8	2	23.2	8.57
W05	GCR	8/12/15	Dry	28	2	2.8	2	23.2	8.57
W06	GCR	8/12/15	Dry	22	2	2.7	2	23.2	8.57
W07	GCE	8/12/15	Dry	0	2	1.1	2	23.2	8.57
W08	GCE	8/12/15	Dry	0	2	0.6	2	23.2	8.57
W09	GCE	8/12/15	Dry	0	2	0.3	2	23.2	8.57
W10	GCM	8/12/15	Dry	4	2	1.6	2	23.2	8.57
W11.1	GCM	8/12/15	Dry	1	2	1.5	2	23.2	8.57
W12	GCM	8/12/15	Dry	4	2	1.4	2	23.2	8.57
W13	GCN	8/12/15	Dry	0	2	0.5	2	23.2	8.57
W14	GCN	8/12/15	Dry	0	2	0.9	2	23.2	8.57
W15	GCN	8/12/15	Dry	0	2	0.4	2	23.2	8.57
W16	JP	8/12/15	Dry	102	2	19.3	4	23.2	8.57
W17	JP	8/12/15	Dry	1230	4	8.0	4	23.2	8.57
W18	JP	8/12/15	Dry	17	2	9.6	4	23.2	8.57
W19	WHW	8/12/15	Dry	12	2	4.8	3	23.2	8.57
W20	WHW	8/12/15	Dry	17	2	4.0	3	23.2	8.57
W21	WHW	8/12/15	Dry	11	2	5.3	3	23.2	8.57
W22	63	9/1/15	Dry	108	2	0.6	2	19.0	10.38
W23	63	9/1/15	Dry	39	2	1.3	2	19.0	10.38
W24	63	9/1/15	Dry	345	3	0.8	2	19.0	10.38
W25	GCR	9/1/15	Dry	12	2	3.0	2	21.8	7.92
W26	GCR	9/1/15	Dry	16	2	3.0	2	21.8	7.92
W27	GCR	9/1/15	Dry	12	2	3.6	3	21.8	7.92
W28	GCE	9/1/15	Dry	12	2	0.5	2	21.8	7.92
W29	GCE	9/1/15	Dry	6	2	1.2	2	21.8	7.92
W30	GCE	9/1/15	Dry	14	2	1.3	2	21.8	7.92
W31	GCM	9/1/15	Dry	3	2	2.0	2	21.8	7.92
W32	GCM	9/1/15	Dry	1	2	2.2	2	21.8	7.92
W33	GCM	9/1/15	Dry	1	2	2.7	2	21.8	7.92
W34	GCN	9/1/15	Dry	2	2	1.6	2	21.8	7.92
W35.1	GCN	9/1/15	Dry	365	3	1.5	2	21.8	7.92
W36	GCN	9/1/15	Dry	99	2	1.5	2	21.8	7.92
W37	JP	9/1/15	Dry	1	2	0.5	2	21.8	7.92
W38	JP	9/1/15	Dry	1	2	1.3	2	21.8	7.92
W39	JP	9/1/15	Dry	0	2	0.5	2	21.8	7.92
W40	WHW	9/1/15	Dry	11	2	1.7	2	21.8	7.92

W41	WHW	9/1/15	Dry	2	2	1.2	2	21.8	7.92
W42	WHW	9/1/15	Dry	4	2	1.9	2	21.8	7.92
W43	63	9/21/15	Wet	46	2	6.5	4	17.7	9.40
W44	63	9/21/15	Wet	167	3	8.9	4	17.7	9.40
W45	63	9/21/15	Wet	47	2	8.0	4	17.7	9.40
W46	GCR	9/21/15	Wet	1034	4	5.1	3	18.3	9.40
W47	GCR	9/21/15	Wet	1633	4	5.0	3	18.3	9.40
W48	GCR	9/21/15	Wet	1226	4	4.5	3	18.3	9.40
W49	GCE	9/21/15	Wet	0	2	2.1	2	18.3	9.40
W50	GCE	9/21/15	Wet	0	2	1.9	2	18.3	9.40
W51	GCE	9/21/15	Wet	0	2	2.1	2	18.3	9.40
W52	GCM	9/21/15	Wet	46	2	2.8	2	18.3	9.40
W53	GCM	9/21/15	Wet	33	2	3.1	2	18.3	9.40
W54	GCM	9/21/15	Wet	31	2	3.3	3	18.3	9.40
W55	GCN	9/21/15	Wet	1	2	1.9	2	18.3	9.40
W56	GCN	9/21/15	Wet	0	2	2.1	2	18.3	9.40
W57	GCN	9/21/15	Wet	1	2	1.8	2	18.3	9.40
W58	JP	9/21/15	Wet	190	3	4.0	3	18.3	9.40
W59	JP	9/21/15	Wet	30	2	4.6	3	18.3	9.40
W60	JP	9/21/15	Wet	10	2	3.8	3	18.3	9.40
W61	WHW	9/21/15	Wet	2	2	5.2	3	18.3	9.40
W62	WHW	9/21/15	Wet	5	2	6.0	4	18.3	9.40
W63	WHW	9/21/15	Wet	4	2	4.6	3	18.3	9.40

Table S6: Significant Spearman rho correlations *E. coli* most probable numbers (MPN) binned and relative abundances of bacteria at genus level

Taxa	Test stat.	p value	p value FDR	p value bonferroni
k_Bacteria;p_Firmicutes;c_Bacilli;o_Lactobacillales;f_Enterococcaceae;g_Enterococcus	0.67902	0.0000	0.0000	0.0000
k_Bacteria;p_TM7;c_TM7-3;o_;f_;g_	0.62775	0.0000	0.0000	0.0000
k_Bacteria;p_Firmicutes;c_Bacilli;o_Bacillales;f_Bacillaceae;g_Anoxybacillus	0.62758	0.0000	0.0000	0.0000
k_Bacteria;p_Proteobacteria;c_Alphaproteobacteria;o_Rhizobiales;f_Beijerinckiaceae;g_Chelatococcus	0.62758	0.0000	0.0000	0.0000
k_Bacteria;p_Proteobacteria;c_Alphaproteobacteria;o_Rhizobiales;f_Hyphomicrobiaceae;Other	0.61717	0.0000	0.0000	0.0000
k_Bacteria;p_Actinobacteria;c_Coriobacteriia;o_Coriobacteriales;f_Coriobacteriaceae;g_Collinsella	0.50827	0.0000	0.0000	0.0000
k_Bacteria;p_Firmicutes;c_Clostridia;o_Clostridiales;f_Ruminococcaceae;g_Faecalibacterium	0.50827	0.0000	0.0000	0.0000
k_Bacteria;p_Firmicutes;c_Clostridia;o_Clostridiales;f_Veillonellaceae;g_Selenomonas	0.50827	0.0000	0.0000	0.0000
k_Bacteria;p_Bacteroidetes;c_Bacteroidia;o_Bacteroidales;f_Rikenellaceae;g_	0.50820	0.0000	0.0000	0.0000
k_Bacteria;p_Firmicutes;c_Clostridia;o_Clostridiales;f_Lachnospiraceae;g_Blautia	0.50820	0.0000	0.0000	0.0000
k_Bacteria;p_Firmicutes;c_Clostridia;o_Clostridiales;Other;Other	0.49021	0.0000	0.0000	0.0000
k_Bacteria;p_Fibrobacteres;c_Fibrobacteria;o_Fibrobacterales;f_;g_	0.48444	0.0000	0.0000	0.0000
k_Bacteria;p_TM6;c_F38;o_;f_;g_	0.46091	0.0000	0.0000	0.0000
k_Bacteria;p_Chlorobi;c_BSV26;o_VC38;f_;g_	0.45313	0.0000	0.0000	0.0000
k_Bacteria;p_Bacteroidetes;c_Bacteroidia;o_Bacteroidales;f_Porphyromonadaceae;g_	0.45186	0.0000	0.0000	0.0000
k_Bacteria;p_Bacteroidetes;c_Bacteroidia;o_Bacteroidales;f_Bacteroidaceae;g_Bacteroides	0.45047	0.0000	0.0000	0.0000
k_Bacteria;p_TM7;c_SC3;o_;f_;g_	0.43739	0.0000	0.0000	0.0000
k_Bacteria;p_Bacteroidetes;c_Cytophagia;o_Cytophagales;f_Cytophagaceae;Other	0.43265	0.0000	0.0000	0.0000
k_Bacteria;p_Proteobacteria;c_Gammaproteobacteria;o_Oceanospirillales;f_Oleiphilaceae;g_	0.42545	0.0000	0.0000	0.0000
k_Bacteria;p_Chloroflexi;c_Chloroflexi;o_Chloroflexales;f_Chloroflexaceae;Other	0.41720	0.0000	0.0000	0.0000
k_Bacteria;p_Proteobacteria;c_Gammaproteobacteria;o_Alteromonadales;f_Alteromonadaceae;g_Cellvibrio	0.41481	0.0000	0.0000	0.0000
k_Bacteria;p_Proteobacteria;c_Gammaproteobacteria;o_Methylococcales;f_Crenotrichaceae;g_Crenothrix	0.41060	0.0000	0.0000	0.0000
k_Bacteria;p_Proteobacteria;c_Betaproteobacteria;o_Burkholderiales;f_Comamonadaceae;g_Tepidimonas	0.40585	0.0000	0.0000	0.0000
k_Bacteria;p_Proteobacteria;c_Alphaproteobacteria;o_Rhizobiales;f_Hyphomicrobiaceae;g_Rhodoplanes	0.40247	0.0000	0.0000	0.0000
k_Bacteria;p_Bacteroidetes;c_Bacteroidia;o_Bacteroidales;f_Porphyromonadaceae;g_Paludibacter	0.40017	0.0000	0.0000	0.0000
k_Bacteria;p_Fusobacteria;c_Fusobacteriia;o_Fusobacteriales;f_;g_	0.39509	0.0000	0.0000	0.0000
k_Bacteria;p_Fibrobacteres;c_;o_;f_;g_	0.39026	0.0000	0.0000	0.0000
k_Bacteria;p_Proteobacteria;c_Gammaproteobacteria;o_Alteromonadales;f_[Chromatiaceae];g_Rheinheimera	0.38623	0.0000	0.0000	0.0000
k_Bacteria;p_Firmicutes;c_Clostridia;o_Clostridiales;f_Clostridiaceae;g_Clostridium	0.38492	0.0000	0.0000	0.0000
k_Bacteria;p_Proteobacteria;c_Gammaproteobacteria;o_Enterobacteriales;f_Enterobacteriaceae;g_	0.36380	0.0000	0.0000	0.0000

k_Bacteria;p_Proteobacteria;c_Epsilonproteobacteria;o_Campylobacterales;f_Helicobacteraceae;g_Sulfurimonas	0.36123	0.0000	0.0000	0.0000
k_Bacteria;p_TM7;c_TM7-1;o_;f_;g_	0.36062	0.0000	0.0000	0.0000
k_Bacteria;p_Firmicutes;c_Bacilli;o_Lactobacillales;f_Streptococcaceae;g_Lactococcus	0.35710	0.0000	0.0000	0.0000
k_Bacteria;p_Proteobacteria;c_Alphaproteobacteria;o_Rhodobacterales;f_Rhodobacteraceae;g_Paracoccus	0.35687	0.0000	0.0000	0.0000
k_Bacteria;p_Actinobacteria;c_Actinobacteria;o_Actinomycetales;f_Cellulomonadaceae;g_Demequina	0.35514	0.0000	0.0000	0.0000
k_Bacteria;p_Bacteroidetes;c_Flavobacteriia;o_Flavobacteriales;f_[Weeksellaceae];g_Cloacibacterium	0.35241	0.0000	0.0000	0.0000
k_Bacteria;p_Spirochaetes;c_Spirochaetes;o_Spirochaetales;f_Spirochaetaceae;g_Treponema	0.35018	0.0000	0.0000	0.0000
k_Bacteria;p_Proteobacteria;c_Betaproteobacteria;o_SBla14;f_;g_	0.34809	0.0000	0.0000	0.0000
k_Bacteria;p_TM6;c_SJA-4;o_;f_;g_	0.33812	0.0000	0.0000	0.0000
k_Bacteria;p_Proteobacteria;c_Deltaproteobacteria;o_Syntrophobacterales;f_Syntrophaceae;g_	0.33712	0.0000	0.0000	0.0000
k_Bacteria;p_Proteobacteria;c_Epsilonproteobacteria;o_Campylobacterales;f_Campylobacteraceae;g_Sulfurospirillum	0.33651	0.0000	0.0000	0.0000
k_Bacteria;p_Proteobacteria;c_Gammaproteobacteria;o_Aeromonadales;f_Aeromonadaceae;g_Tolumonas	0.33638	0.0000	0.0000	0.0000
k_Bacteria;p_Bacteroidetes;c_Bacteroidia;o_Bacteroidales;f_BA008;g_	0.33602	0.0000	0.0000	0.0000
k_Bacteria;p_Bacteroidetes;c_[Saprospirae];o_[Saprospirales];f_Saprospiraceae;g_	0.32427	0.0000	0.0000	0.0000
k_Bacteria;p_Proteobacteria;c_Betaproteobacteria;o_ASSO-13;f_;g_	0.32170	0.0000	0.0000	0.0000
k_Bacteria;p_Firmicutes;c_Clostridia;o_Clostridiales;f_[Mogibacteriaceae];g_Anaerovorax	0.32140	0.0000	0.0000	0.0000
k_Bacteria;p_Firmicutes;c_Clostridia;o_Clostridiales;f_Gracilibacteraceae;g_	0.32001	0.0000	0.0000	0.0000
k_Bacteria;p_Elusimicrobia;c_Elusimicrobia;o_Elusimicrobiales;f_;g_	0.30763	0.0000	0.0000	0.0000
k_Bacteria;p_Actinobacteria;c_OPB41;o_;f_;g_	0.30709	0.0000	0.0000	0.0000
k_Bacteria;p_Firmicutes;c_Clostridia;o_Clostridiales;f_[Mogibacteriaceae];g_	0.29945	0.0000	0.0000	0.0000
k_Bacteria;p_Proteobacteria;c_Betaproteobacteria;o_Nitrosomonadales;f_Nitrosomonadaceae;g_	0.28985	0.0000	0.0000	0.0000
k_Bacteria;p_Proteobacteria;c_Betaproteobacteria;o_Rhodocyclales;f_Rhodocyclaceae;g_Zoogloea	0.27324	0.0000	0.0000	0.0000
k_Bacteria;p_Proteobacteria;c_Alphaproteobacteria;o_Sphingomonadales;f_Sphingomonadaceae;g_	-0.30628	0.0000	0.0000	0.0000
